# Supplementary material for: Oncolytic Group B Adenovirus Enadenotucirev Mediates Non-apoptotic Cell Death with Membrane Disruption and Release of Inflammatory Mediators
Source: Mol Ther Oncolytics. 2016 Dec 10;4:18–30. doi: 10.1016/j.omto.2016.11.003 (PMC5363721; doi:10.1016/j.omto.2016.11.003)
Supplement: Document S1. Figures S1–S7 and Table S1 [file mmc1.pdf]

OMTO, Volume *4*

Supplemental Information

Oncolytic Group B Adenovirus Enadenotucirev Mediates Non-apoptotic Cell Death with Membrane Disruption and Release of Inflammatory Mediators

Arthur Dyer, Ying Di, Hugo Calderon, Sam Illingworth, Gray Kueberuwa, Alison Tedcastle, Phil Jakeman, Suet Lin Chia, Alice Brown, Michael A. Silva, David Barlow, John Beadle, Terry Hermiston, David J.P. Ferguson, Brian Champion, Kerry D. Fisher, and Leonard W. Seymour

Table 1. Particle number and infectious titre of virus stocks

	Particle number/ml (VP/ml)*	Infectious particle number/ml (PFU/ml)*	**VP/PFU ratio**
Ad5	2.50E12	7.07E10	**35.3**
Ad5 (fig 1J, fig 5 and fig 6E)	3.16E11	1.05E10	**30.1**
Ad11	1.90E12	6.13E10	**31.0**
Ad11 (fig 1K, fig 5 and fig 6E)	2.35E12	7.15E10	**32.9**
EnAd	1.65E12	4.77E10	**34.6**
EnAd (fig 1L, fig 5 and fig 6E)	1.69E12	4.81E10	**35.1**
Ad3	1.45E12	ND	**ND**
ONYX-15	4.88E12	ND	**ND**

*VP/ml was measured by Picogreen assay and PFU/ml was measured by 7 days TCID50 assay on A549 cells.

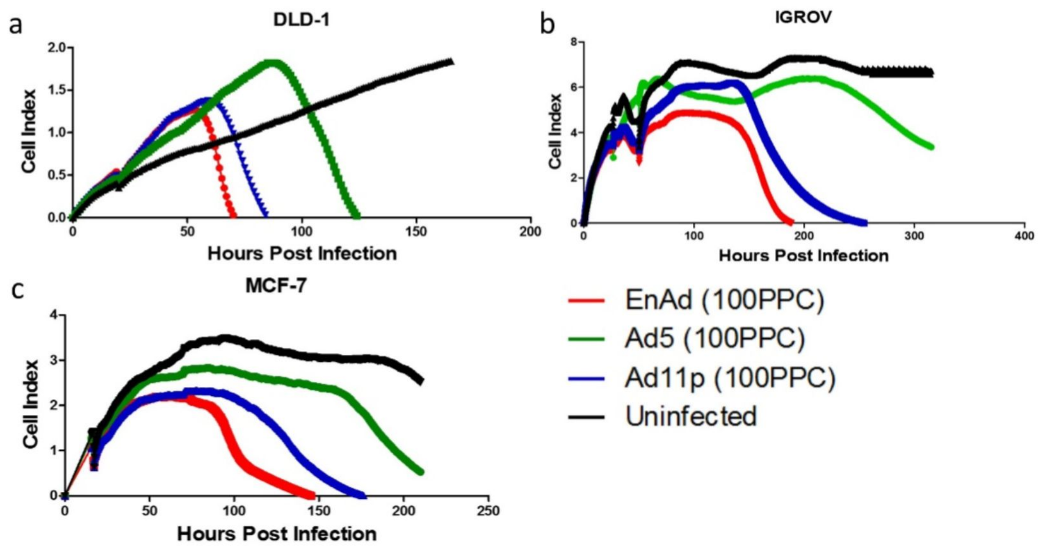

Supplementary figure S1 - Comparison of potency and time course of cytotoxicity of different adenoviruses in different cell lines. DLD-1 **(a)** IGROV **(b)** and MCF-7 **(c)** cell growth and cytotoxicity curve under 100PPC of different adenoviruses, measured by xCelligence cell monitor (Roche)**.**

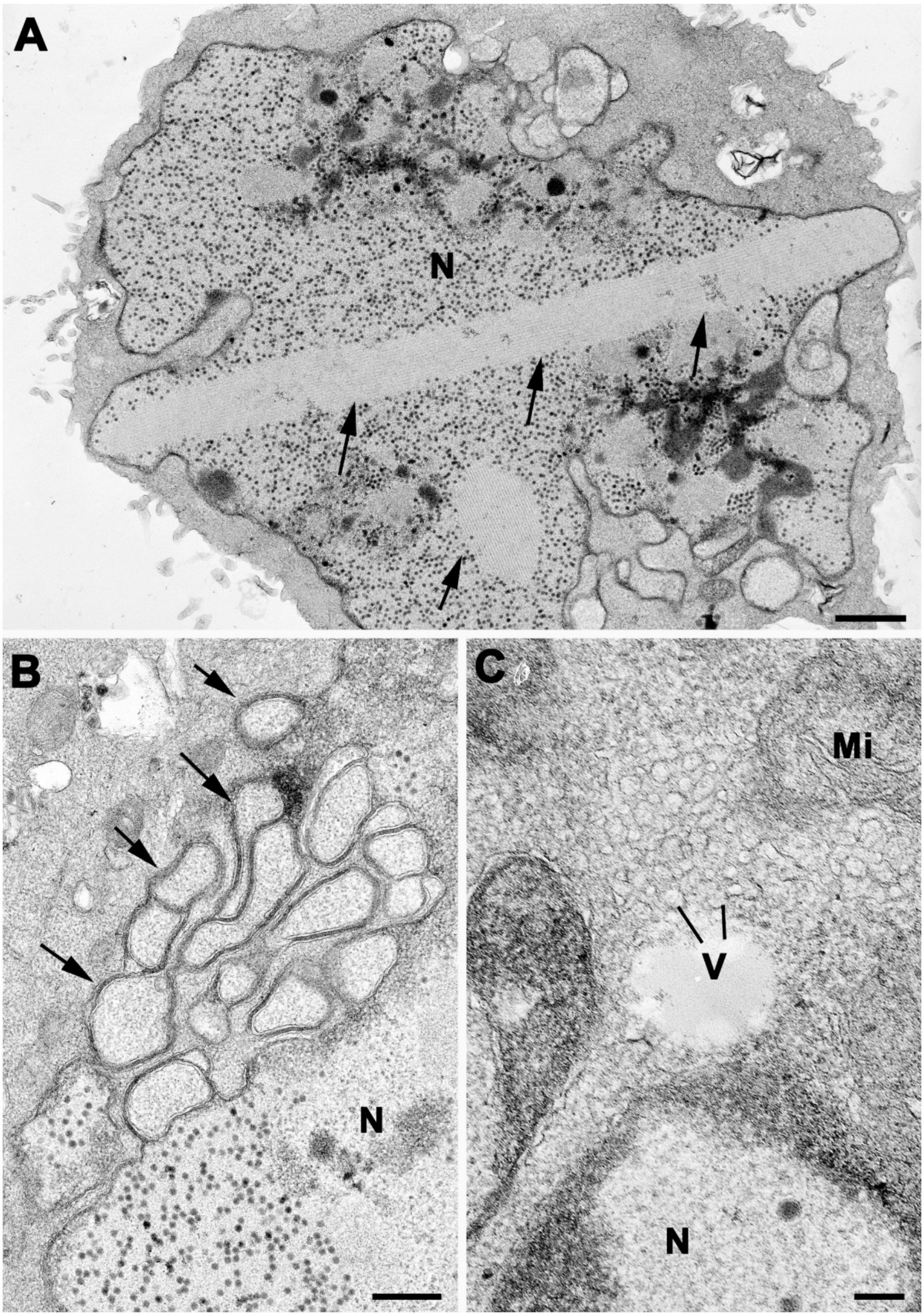

Supplementary Figure S2. Electron micrographs of non-physiological features observed in A549 cells infected with Ad5. Arrows in **A** indicate intranuclear crystals of virus proteins, in **B** excessive folding of the nuclear membrane and in **C** the presence of many vesicles or membranes in the cytoplasm (V). N= nucleus, Mi=mitochondrion. The bars in A - 1um, B - 500nm and C -100nm.

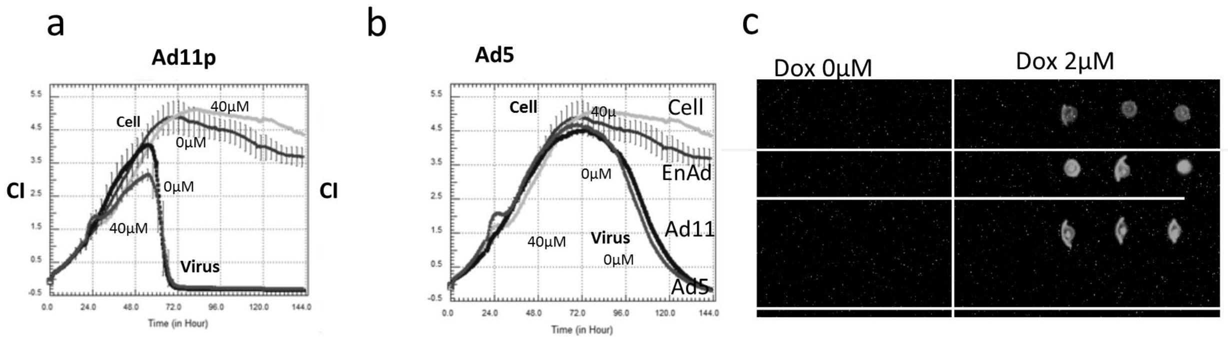

Supplementary Figures S3 a&b The impact of p53 inhibitor Pitfithrin α on activity of Ad5 and Ad11p in A549 cells. Cell growth was assessed using the Xcelligence system as described, adenoviruses were applied at 100 PPC, 24h after cell seeding (i.e. at 24h on the graph). Pitfithrin α was applied at 40 μM, 5 h after viral infection. **c**, Dot blot for p53 of the samples obtained from Fig3 **c** to verify p53 induction by doxycycline. Cells were lysed using 50μl Promega lysis buffer, and 1μl lysate from each well was assayed.

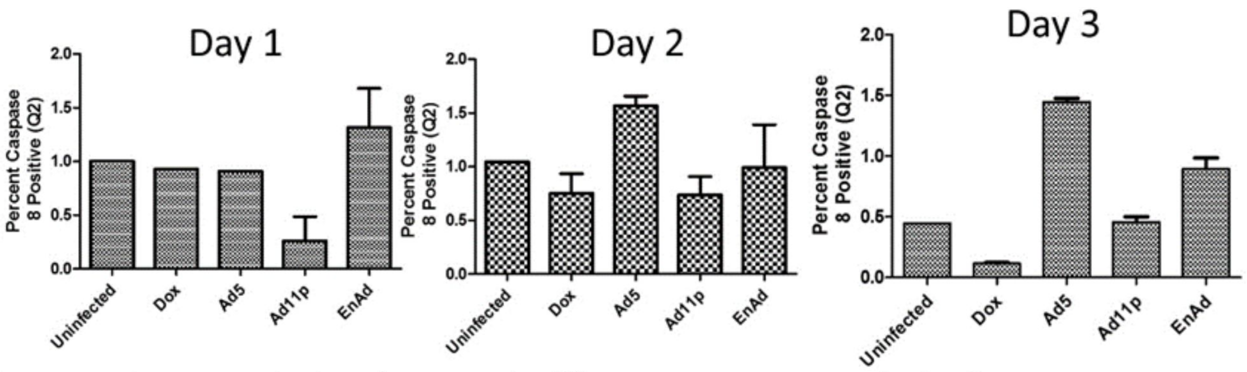

Supplementary Figure S4. Activation of caspase 8 by different treatments, assessed using flow cytometry. Activation of caspase 8 in A549 cells following treatment with viruses (100 ppc) or doxorubicin (5 mM). Cells were harvested at 24, 48 and 72h and assayed using the Vybrant FAM Caspase-8 assay kit.

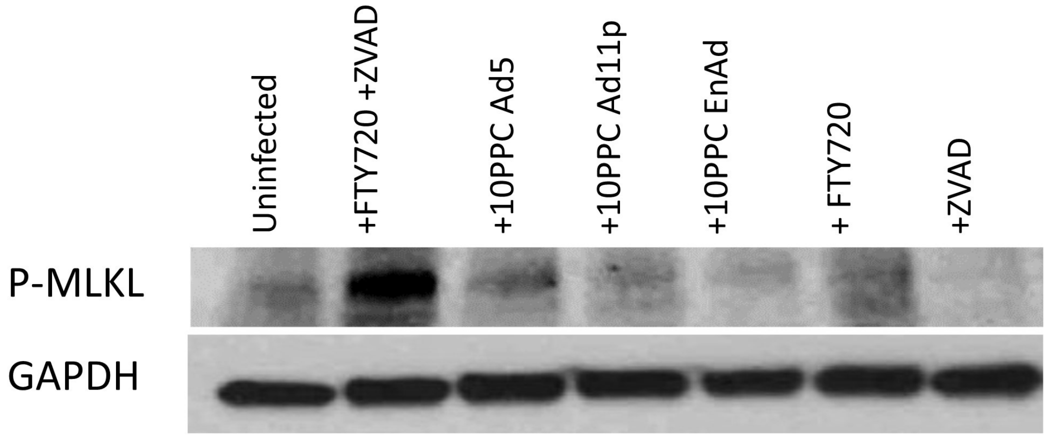

Supplementary Figure S5. Presence of markers of necroptosis during infection with adenoviruses

Western blot analysis of Phospho-MLKL (Ser358) in A549 Cells treated with different viruses and drugs. Cells were treated with 20mMol FTY720, 20µMol ZVAD, a combination of both FTY720 and ZVAD or 10PPC of Ad5, Ad11p or EnAd. 4 days post treatment cells were lysed and analysed via western blot for phospho-MLKL (Ser358) and GAPDH.

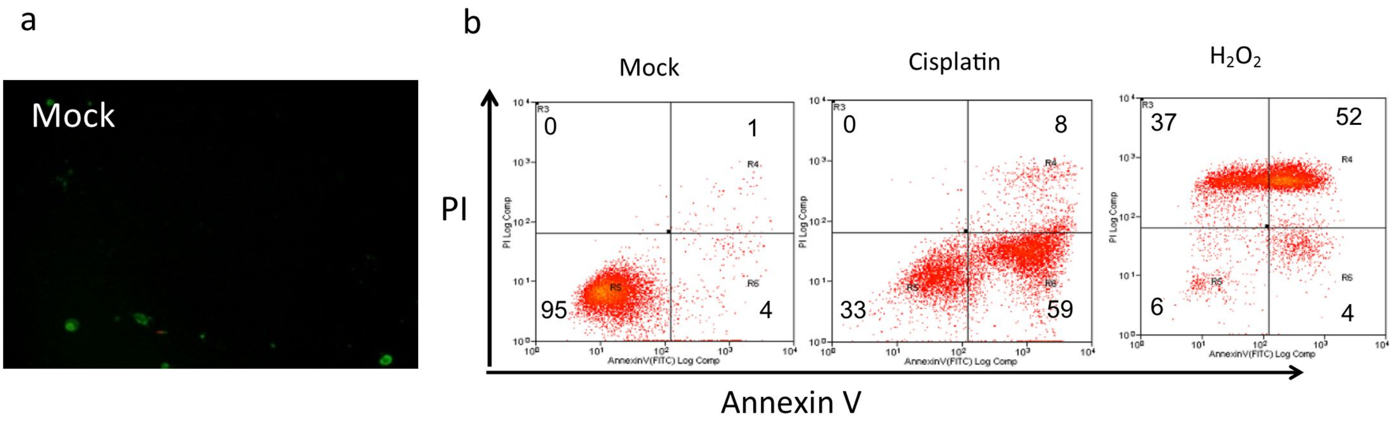

Supplementary Figure S6a. AnnexinV/PI dual staining of non-treated cells. Cells were observed by fluorescence microscopy, with 10X magnification**.** Annexin V was labelled with FITC (green color); PI was shown as red colour. **b**, **Representive Annexin V/PI double staining dot blot analysis by flow cytometry.** Cisplatin was applied at 50µM for 24 h, and H_2O_2 was used at 1mM for 3 h.

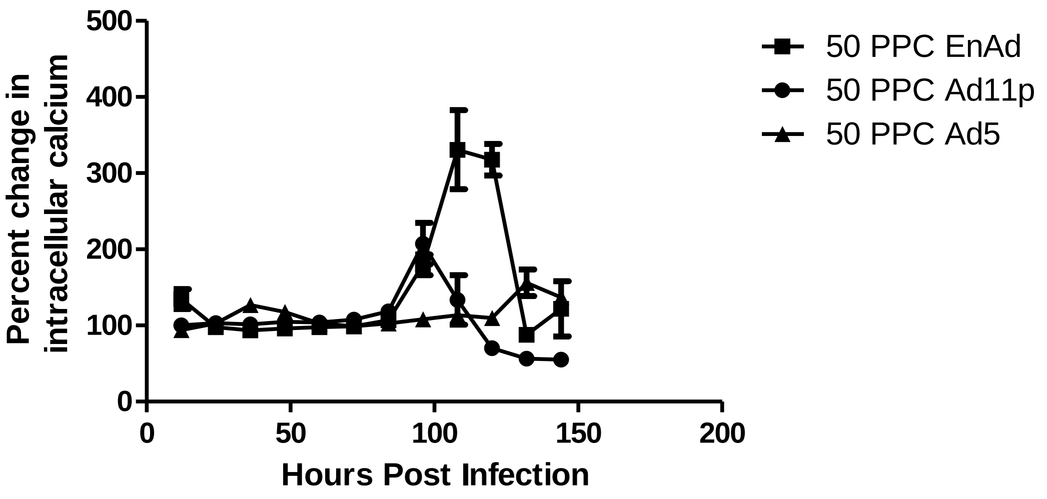

Supplementary figure S7 Effects of EnAd, Ad5 and Ad11p infection and replication on calcium levels in A549 Cells. Intracellular calcium levels were measured in A549 Cells and expressed as % of the initial value
