# Supplementary figures and images for: Oncolytic Group B Adenovirus Enadenotucirev Mediates Non-apoptotic Cell Death with Membrane Disruption and Release of Inflammatory Mediators
Source: Mol Ther Oncolytics. 2016 Dec 10;4:18–30. doi: 10.1016/j.omto.2016.11.003 (PMC5363721; doi:10.1016/j.omto.2016.11.003)

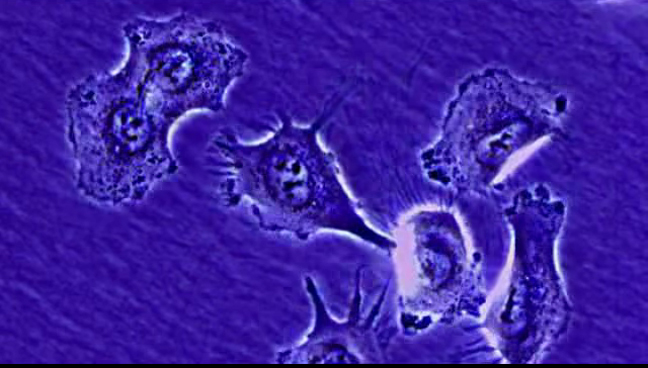

Supplement: Movie S1. Time Lapse Video Microscopy A549 Cells Exposed to Cisplatin [file mmc2.jpg]

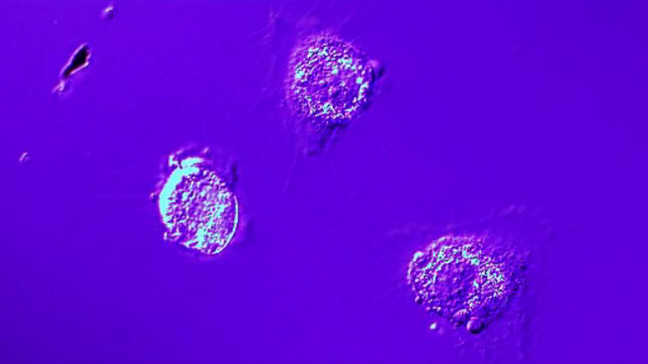

Supplement: Movie S2. Time Lapse Video Microscopy A549 Cells Exposed to Hydrogen Peroxide [file mmc3.jpg]

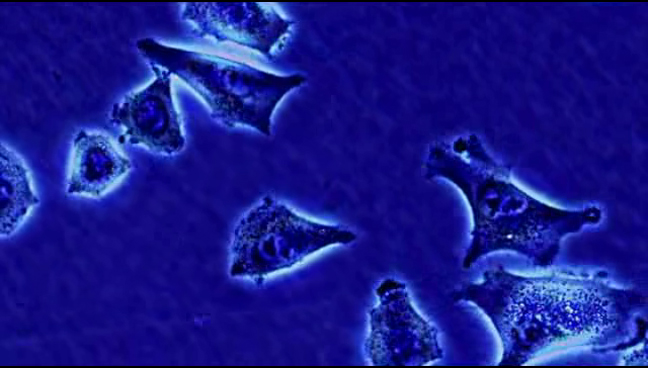

Supplement: Movie S3. Time Lapse Video Microscopy A549 Cells Exposed to Wild-Type Adenovirus 5 [file mmc4.jpg]

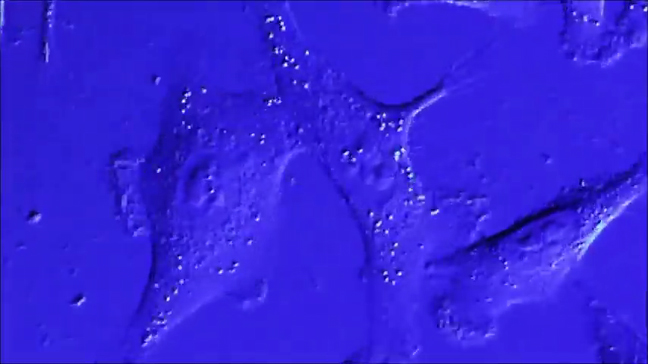

Supplement: Movie S4. Time Lapse Video Microscopy A549 Cells Exposed to EnAd [file mmc5.jpg]

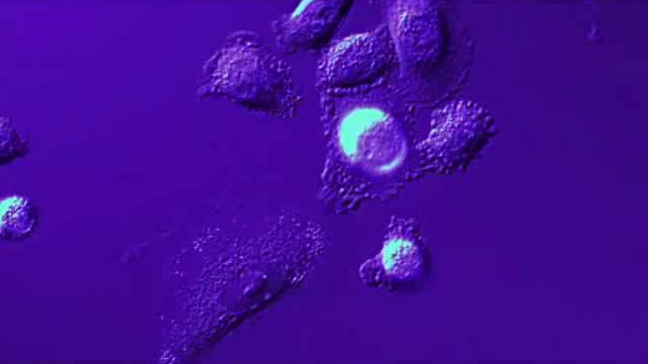

Supplement: Movie S5. Time Lapse Video Microscopy A549 Cells Exposed to Wild-Type Adenovirus 11p [file mmc6.jpg]
