# Supplementary material for: Oncolytic Group B Adenovirus Enadenotucirev Mediates Non-apoptotic Cell Death with Membrane Disruption and Release of Inflammatory Mediators
Source: Mol Ther Oncolytics. 2016 Dec 10;4:18–30. doi: 10.1016/j.omto.2016.11.003 (PMC5363721; doi:10.1016/j.omto.2016.11.003)
Supplement: Document S2. Article plus Supplemental Information [file mmc7.pdf]

Molecular Therapy
Oncolytics
Original Article

AMERICAN SOCIETY of GENE & CELL THERAPY

Oncolytic Group B Adenovirus Enadenotucirev Mediates Non-apoptotic Cell Death with Membrane Disruption and Release of Inflammatory Mediators

Arthur Dyer,[1,6] Ying Di,[1,6] Hugo Calderon,[2] Sam Illingworth,[2] Gray Kueberuwa,[1] Alison Tedcastle,[1] Phil Jakeman,[1] Suet Lin Chia,[1] Alice Brown,[2] Michael A. Silva,[3] David Barlow,[1] John Beadle,[2] Terry Hermiston,[4] David J.P. Ferguson,[5] Brian Champion,[2] Kerry D. Fisher,[1] and Leonard W. Seymour[1]

[1]Department of Oncology, University of Oxford, Oxford OX3 7DQ, UK; [2]PsiOxus Therapeutics, Ltd., Milton Park, Abingdon OX14 4SD, UK; [3]Department of Surgical Sciences, University of Oxford, Oxford OX3 9DU, UK; [4]Bayer HealthCare, 455 Mission Bay Blvd. S., San Francisco, CA 94158, USA; [5]Nuffield Department of Clinical Laboratory Science, University of Oxford, Oxford OX3 9DU, UK

Enadenotucirev (EnAd) is a chimeric group B adenovirus isolated by bioselection from a library of adenovirus serotypes. It replicates selectively in and kills a diverse range of carcinoma cells, shows effective anticancer activity in preclinical systems, and is currently undergoing phase I/II clinical trials. EnAd kills cells more quickly than type 5 adenovirus, and speed of cytotoxicity is dose dependent. The EnAd death pathway does not involve p53, is predominantly caspase independent, and appears to involve a rapid fall in cellular ATP. Infected cells show early loss of membrane integrity; increased exposure of calreticulin; extracellular release of ATP, HSP70, and HMGB1; and influx of calcium. The virus also causes an obvious single membrane blister reminiscent of ischemic cell death by oncosis. In human tumor biopsies maintained in ex vivo culture, EnAd mediated release of pro-inflammatory mediators such as TNF-α, IL-6, and HMGB1. In accordance with this, EnAd-infected tumor cells showed potent stimulation of dendritic cells and $CD4^+$ T cells in a mixed tumor-leukocyte reaction in vitro. Whereas many viruses have evolved for efficient propagation with minimal inflammation, bioselection of EnAd for rapid killing has yielded a virus with a short life cycle that combines potent cytotoxicity with a proinflammatory mechanism of cell death.

INTRODUCTION

Cancer virotherapy exploits the ability of lytic viruses to replicate selectively within cancer cells and lyse them before spreading to infect adjacent cells.[1,2] Recent advances in cancer biology have enabled molecular engineering of viruses to exploit specific acquired features of the cancer genotype or phenotype, with some agents showing encouraging clinical success.[3] Notably, following the announcement that Amgen's oncolytic herpes vaccine had met its primary endpoint of improved durable response rate in a phase 3 melanoma trial,[4] it has been awarded a product license in both the United States and European Union. Rather than design the molecular structure of viruses to exploit known mutations, we have used a bioselection process to isolate chimeric adenoviruses with the desired biological properties from a diverse library of adenovirus serotypes under conditions designed to encourage recombination.[5] One such chimeric adenovirus, known as Enadenotucirev or EnAd (formerly known as "ColoAd1") shows particular potency for killing cancer cells while sparing normal cells both in vitro and in vivo,[5] and is currently undergoing a series of early-phase clinical trials.

The capsid of EnAd is from Ad11p, a serotype with limited seroprevalence in humans. EnAd infects cells by binding to CD46 and/or desmoglein 2,6 both widely expressed on many carcinoma cells. Most of the EnAd genome is derived from Ad11p with a large deletion in E3 and a smaller deletion in E4. In addition, the E2B region consists of a chimera of sequences from Ad11p and Ad3.[5] Adenovirus E3 proteins are generally thought to protect virally infected cells from eradication by the immune system, although their precise functions in group B viruses are not fully delineated. The E4 deletion in EnAd is in E4ORF4, which in Ad5 encodes a protein that inactivates protein phosphatase2A and thereby activates protein translation machinery as well as regulating activity of E1A protein in a feedback inhibitory loop.[7] These deletions, perhaps combined with the chimeric E2B region, probably contribute to the striking cancer-selective replication of EnAd.[5]

Many viruses kill cells by activating apoptosis mechanisms, although the possibility that some viruses mediate lysis without apoptosis is attractive because it may provide a pro-inflammatory environment useful in assisting a cancer vaccine strategy. Here, we characterize the cytotoxicity of EnAd, showing that the virus kills cells more quickly than wild-type Ad3, Ad11p, and Ad5, largely independent

Received 19 July 2016; accepted 28 November 2016;
http://dx.doi.org/10.1016/j.omto.2016.11.003.

[6]These authors contributed equally to this work.

Correspondence: Leonard W. Seymour, Department of Oncology, University of Oxford, Old Road Campus Research Building, Old Road Campus off Roosevelt Drive, Headington, Oxford, Oxon OX3 7DQ, UK.
E-mail: len.seymour@oncology.ox.ac.uk

 © 2016 The Author(s).
This is an open access article under the CC BY license (http://creativecommons.org/licenses/by/4.0/).
CrossMark

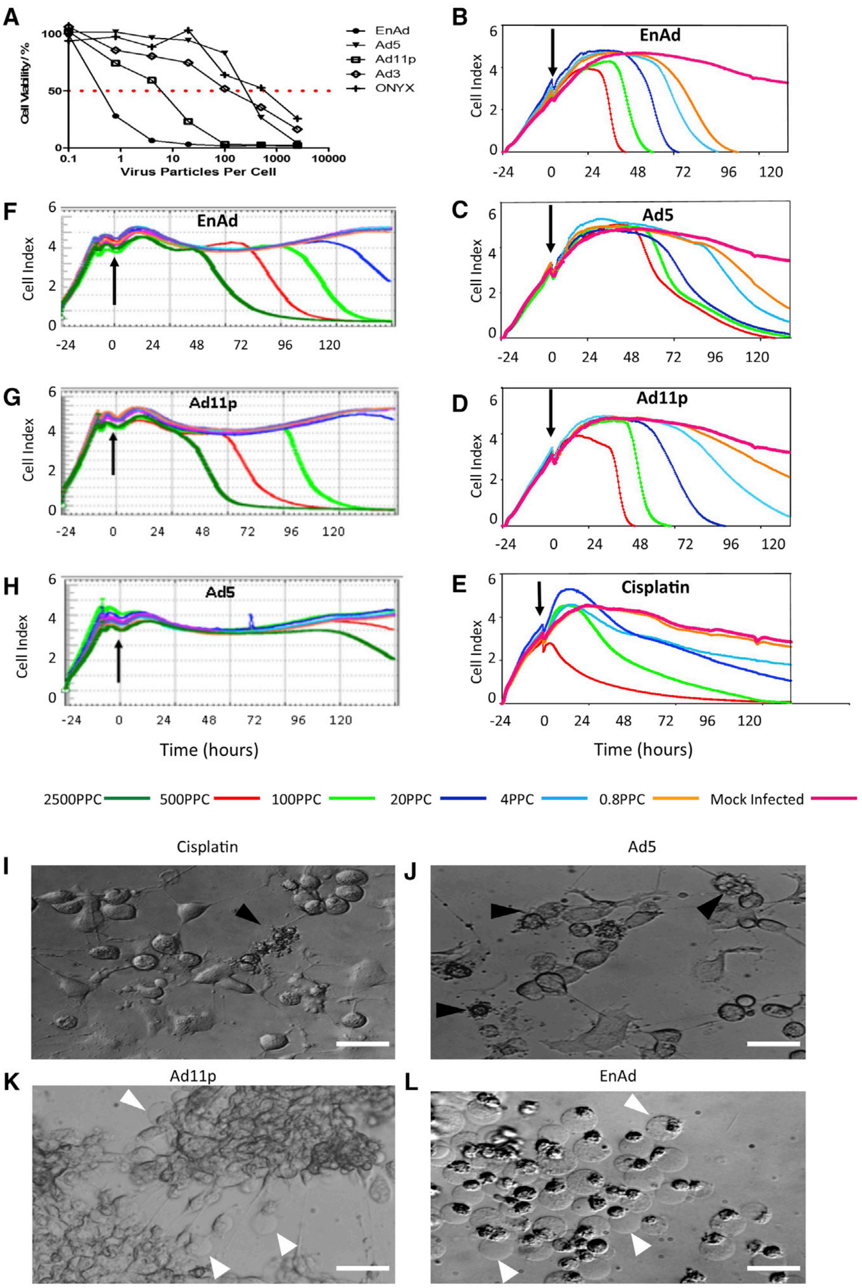

Figure 1. Comparison of Potency and Time Course of Cytotoxicity of Different Adenoviruses in A549 Cells

(A) A549 cells were exposed to EnAd, Ad5, Ad3, Ad11p, and Onyx-015 at different ppc, and viability was measured by MTS assay over 5 days. The dashed line shows 50% cell survival, indicating IC_{50} values. (B–D) A549 cell growth and cytotoxicity curve under titrated doses of different adenoviruses, measured by xCELLigence cell monitor (Roche). The color of the lines refers to the virus dose shown (see key). (E) A549 cell growth and cytotoxicity curve under titrated doses of cisplatin (500, 100, 20, 4.0, 0.8, and 0 μM, respectively, using the same rank order color code as B–D). (F–H) Cell growth and cytotoxicity graphs for SKOV-3 ovarian carcinoma cells, using the same virus concentrations and color codes as (B)–(D), but with an additional high dose (dark green lines) of 2,500 ppc. In all cases, the vertical arrow shows when the treatment was added. (I–L) Light microscopy images of A549 cells exposed to different cytotoxic treatments; cisplatin (100 μM), Ad5, Ad11p, and EnAd (all 100 ppc), and photographed after 48 hr. Scale bar, 100 μm.

of programmed cell death (apoptosis and necroptosis) mechanisms. The death pathway coincides with a rapid fall in cellular ATP and has many features associated with ischemic cell death or oncosis. These include the formation of large single-cell blisters, thought to reflect loss of control of cellular ion gradients. Death by oncosis is usually considered pro-inflammatory, and EnAd-mediated death causes significant release of inflammatory mediators from cells such as ATP, HMGB1, heat shock proteins, and exposure of calreticulin. In a mixed tumor-leukocyte reaction, tumor cells infected with EnAd gave a strong activation of dendritic cells that led in turn to potent activation of CD4 T cells. These features of EnAd infection of cancer cells support the notion that it has a pro-inflammatory phenotype and may enhance the utility of the virus in promoting an anticancer vaccine response.

RESULTS

Enadenotucirev Oncolysis Is Faster and More Potent Than Wild-Type Adenovirus

The cytotoxicity of EnAd at a range of concentrations was compared with three wild-type adenoviruses (Ad5, Ad11p, and Ad3) and the oncolytic adenovirus Onyx-015 on A549 cells (Figure 1A). The virus batches used all had similar infectivity titers on 293T cells (measured by $TCID_{50}$; see Supplemental Information), but in this 5-day cytotoxicity assay EnAd was far more potent than the other viruses at all concentrations. Under these conditions, the IC_{50} (the virus concentration achieving 50% cell kill) for EnAd was over 1,000-fold lower than for Ad5, Ad3, and onyx-015, and more than 20-fold lower than for Ad11p.

Cytotoxicity was also compared in real time using the xCELLigence system (Figures 1B–1H), which measures changes in conductivity to indicate growth of the cell monolayer for adherent type cells, giving a cell index (CI) which rises as cells proliferate and falls as they die or detach from the plate.[8] Viability of the remaining cells can also be verified by MTS assay and crystal violet staining. In all cases, viruses were added 24 hr after the start of cell culture, when cells were

growing rapidly. EnAd and Ad11p were noticeably more potent than Ad5 in all cell lines studied. At the highest concentration (500 particles per cell [ppc]) in A549 cells, they mediated complete killing between 36-48 hr post-infection (p.i.; Figures 1B and 1D), with EnAd more active than Ad11p at lower concentrations. In contrast, high doses of Ad5 required 3–5 days to achieve full cell killing (Figure 1C). The "kill" curves caused by the group B adenoviruses (sudden falls in the cell index; Figures 1B and 1D) were roughly parallel at different virus concentrations but began at different times, suggesting that the time of killing was largely dependent on virus dose. In contrast, the kill curves of high doses of Ad5 (Figure 1C) were similar to each other and largely superimposed, indicating that the timing of cell death was less dependent on virus dose. It is possible that the dose-dependent cytotoxicity of EnAd and Ad11p reflects virus-mediated cytotoxicity pathways, whereas the largely dose-independent Ad5 cytotoxicity might indicate more involvement of physiological death pathways. Under the same conditions, cisplatin killed A549 cells over 4–5 days (Figure 1E) typical of programmed cell death. The dose-dependent cytotoxicity of EnAd and Ad11p was not restricted to A549 cells, with similar results obtained in other cells including SKOV-3 (Figures 1F–1H) and DLD-1, IGROV, and MCF-7 cells (Figure S1). Cancer selectivity of EnAd has been extensively characterized using a broad range of human primary cells from a range of tissues (S.I., unpublished data).

Enadenotucirev-Induced Cytotoxicity Shows "Single-Membrane Blister" Death Associated with Oncosis

Cytotoxicity induced by the different treatments was assessed using time-lapse video light microscopy (Movies S1, S2, S3, S4, and S5). Cells treated with Ad5 ceased dividing, became isolated and rounded, and eventually died with excessive multiple membrane blebbing characteristic of apoptosis. Cisplatin also induced death with features of apoptosis. In contrast, cells treated with EnAd and Ad11p usually clumped together, showed little evidence of apoptosis, and underwent sudden formation of large single membrane blisters at the time of death[9] (Movies S1, S2, S3, S4, and S5). This dramatic cellular demise, termed single blister cell death (BCD) is a typical marker of cell death by oncosis[10,11] and is thought to reflect loss of control over ion transport and consequent osmotic rupture. Figures 1I–1L shows individual frame images, taken from the movies, and the single membrane blisters caused by EnAd are clearly visible.

Enadenotucirev-Induced Cytotoxicity Does Not Show the Classical Signs of Apoptosis or Necroptosis

It was unclear whether the BCD death pathway caused by the group B adenoviruses was dependent on classical components of programmed cell death. Accordingly, we explored several features associated with apoptosis and necroptosis to determine any involvement of these processes in adenovirus cytotoxicity.

Cell Morphology

The effects of virus infection and replication on cellular morphology were assessed by transmission electron microscopy (TEM). This was performed late in the virus life cycles, when intranuclear tessellations of capsids were visible in each case, but before obvious cell death. A549 cells infected with Ad5 showed substantial disturbance of cell morphology, including extensions of the nuclear membrane and fragmentation of the nucleus (Figures 2A and S2). In contrast, EnAd and Ad11p infection caused no obvious cytopathology despite the presence of large numbers of virus particles in the nucleus. Classic features of apoptosis, such as cell shrinkage and chromatin condensation, were not seen with any of the viruses.

Cell Cycle

The effects of virus infection on the cell cycle were assessed by flow cytometry at times and conditions when the various agents were exerting maximum cytotoxicity (see Figure 2B legend for details). A549 cells infected with EnAd or Ad11p showed little perturbation of the cell cycle, similar to peroxide-treated cells (Figure 2B), except many cells (up to 20%) showed greater than 2N (>2N) levels of DNA, most likely representing detection of virus DNA alongside the genomic DNA. Cells infected with Ad5 also showed this increase in >2N cells, but in addition they showed a dramatic fall in G_1 and a rise in S phase. These latter features were shared by cells treated with cisplatin, which also caused a major increase in sub-G_1, indicating the formation of apoptotic bodies, although this was not observed with any virus treatment.

Involvement of p53 in Virus Cytotoxicity

To try and elucidate the mechanism of EnAd cytotoxicity, we assessed the possible involvement of p53. A549 cells (which have wild-type p53) were infected with EnAd, Ad5, or Ad11p, and cell lysates were assessed by western blot. Expression of hexon protein was used to show that each virus progressed through its life cycle to the expression of late proteins by day 3. Cells infected with Ad5 showed a small rise in p53 protein (day 1) before a substantial fall (days 2, 3) thought to reflect targeted degradation.[12] In contrast, both group B viruses showed time-dependent increases in p53 (Figure 3A). Ad11 has been shown previously to cause a rise in p53 protein, and it is thought that the increased p53 is transcriptionally inactivated and recruited to viral replication centers.[13]

Pitfithrin-α inhibits transactivation of p53-responsive genes and suppresses p53-dependent apoptosis.[14] When added to A549 cells, studied by xCELLigence, it had little effect on non-infected cells but also had no effect on the cytotoxicity of EnAd (Figure 3B), suggesting p53 is not involved in the EnAd-mediated death mechanism. Similarly, when using a p53-null human lung carcinoma cell line with inducible p53 (H1299-p53-tet-on), induction of p53 using doxycycline (Figure S3) did not affect cytotoxicity of EnAd, Ad11p, or Ad5, although it did increase cisplatin cytotoxicity as expected (Figure 3C). These data confirm that, in this system, p53 does not play an important role in the cytotoxicity of EnAd nor of wild-type Ad11p or Ad5.

Caspase Activation

We then assessed the involvement of caspases 8 and 3 during virus-induced cell death, markers of extrinsic and late apoptosis, respectively.[15] Analysis of caspase 8 activation by flow cytometry of A549

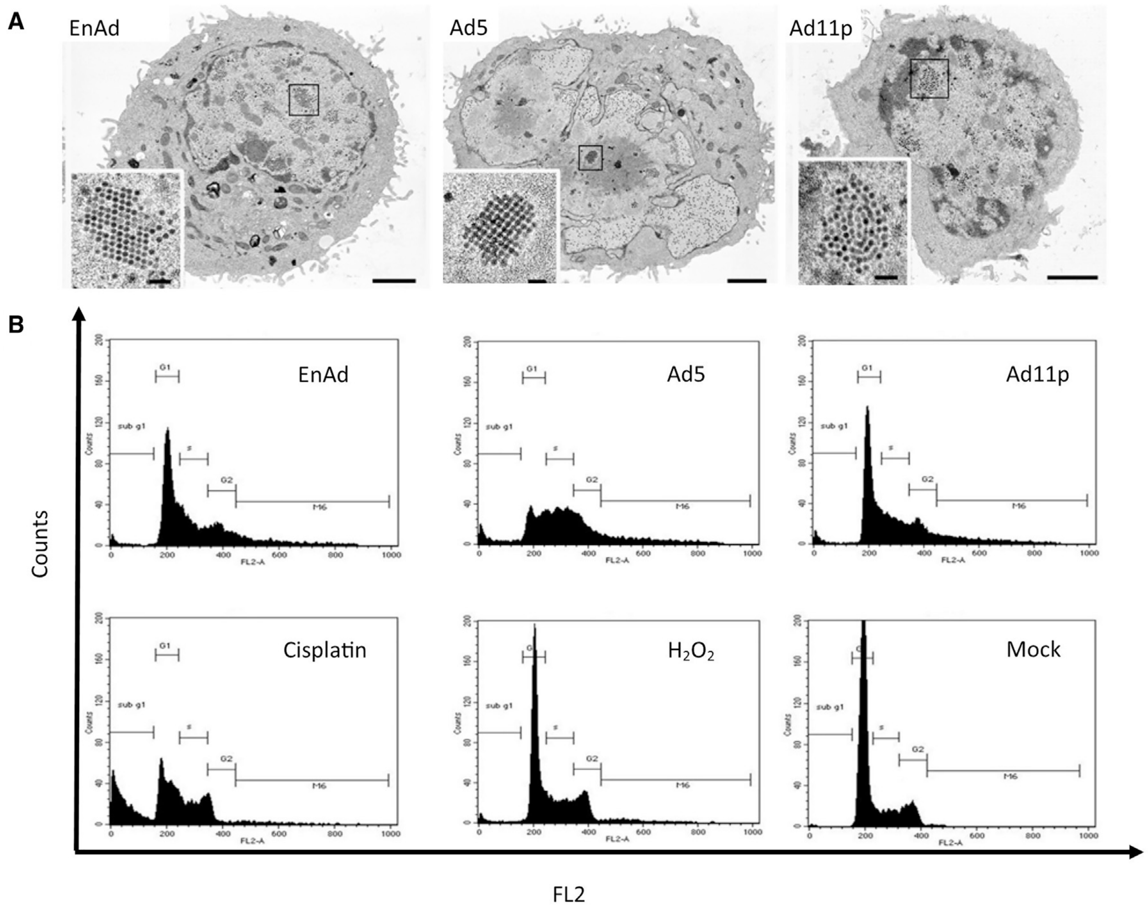

Figure 2. The Impact of Oncolysis on Morphology and Cell Cycle

(A) Following exposure to viruses, the morphology of A549 cells was evaluated by transmission electron microscopy (TEM), comparing cell morphologies at similar stage in the virus life cycle (scale bars show 1 μm in the main images and 100 nm for insets). (B) Cell-cycle analysis by flow cytometry, with cells treated as shown. Treatment conditions applied were as follows: EnAd, 10 ppc (POI day 3); Ad11, 25 ppc (POI day 3), and Ad5, 100 ppc (POI day 3.5); cisplatin (50 μM) assessed after 24 hr, H_2O_2 (1 mM) assessed after 4 hr.

cells showed that none of the viruses caused measurably increased levels during days 1–2 post-infection, but Ad5-treated cells showed activation of caspase 8 day 3 post-infection (Figure S4). This indicates that the extrinsic pathway of apoptosis does not play a role in virus-induced cell death. In contrast, EnAd- and Ad11p-treated cells showed modest levels of caspase 3 activation (approximately 10% increase compared to controls; Figure 3) 24–72 hr following infection, suggesting that a subset of cells may show some features of apoptosis later in the death pathway. Because virtually all of the cells are dead by 72–96 hr, it seems likely that the majority of cells are dying without measurable activation of caspase pathways.

Necroptosis

Apoptosis does not seem to play a major role in adenovirus-mediated cytotoxicity in these systems, leading us to consider the involvement of Rip1-kinase, known to play a central role in pathways of cellular inflammation and programmed necrosis (necroptosis). We therefore evaluated phosphorylation of mixed lineage kinase domain-like protein (MLKL), which plays a key role in death domain-mediated necroptosis, but found no measurable activation by any of the viruses (Figure S5). These data suggest that Rip-1 kinase plays no significant role in the mechanism of EnAd cytotoxicity.

EnAd Oncolysis Shows Features of Oncosis

To investigate whether cells infected with EnAd and Ad11p were undergoing oncosis, we evaluated the expression of typical oncosis markers,[16] including early loss of membrane integrity, a rapid fall in intracellular ATP, and rise in intracellular calcium, as well as single blister formation (see above).[11,17]

Membrane Integrity

Membrane integrity associated with cell death was characterized by Annexin V/PI double staining, allowing differentiation of apoptotic, necrotic, and viable cells (Figures 4A and 4B). Whereas viable cells are unstained, apoptotic cells are positive for Annexin V, reflecting binding to phosphatidylserine, which is externalized, although the

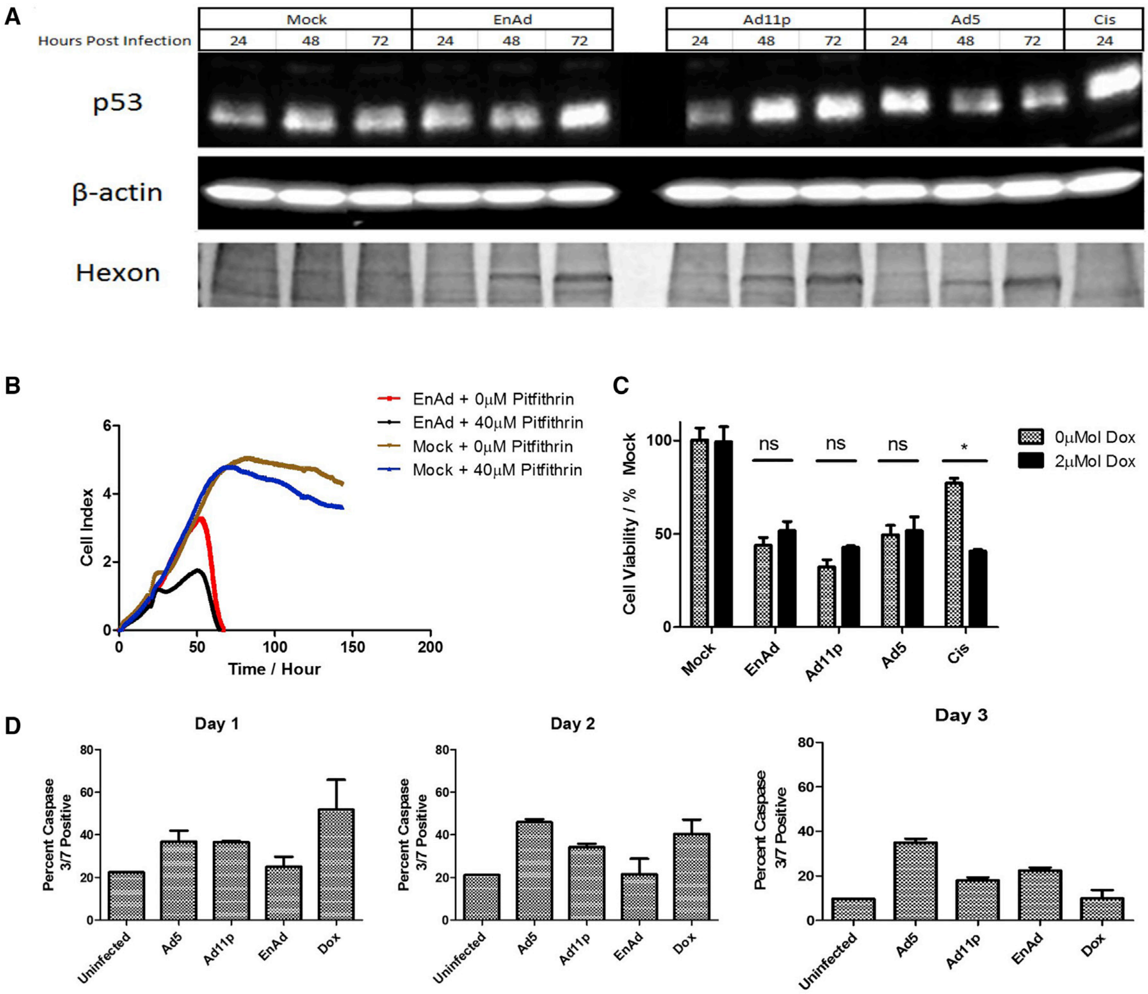

Figure 3. Adenovirus Cytotoxicity Does Not Involve p53 or Caspase 3 Activation

(A) Western blot of p53 levels in A549 cells treated with different viruses (EnAd, 10 ppc; Ad11, 25 ppc; Ad5, 100 ppc) or cisplatin (50 μM). β-Actin as loading control. Viral replication was visualized by Coomassie blue-stained adenovirus hexon bands (molecular weight [MW], 120 kDa approximately). (B) The impact of p53 inhibitor Pitfithrin α on EnAd (100 ppc)-mediated cytotoxicity measured by xCELLigence; cells were seeded at time 0 and virus added at 24 hr. Pitfithin α (40 μM) was applied 5 hr POI. (C) Effect of p53 expression on cytotoxicities of viruses and cisplatin on H1299-p53-Tet on cells, measured by MTS assay 48 hr POI. For viral treatment, doxycycline (2 μM) was added 5 hr POI to induce p53 expression (Figure S3); for cisplatin treatment, doxycycline was added 16 hr before cisplatin. Significance was assessed using two-way ANOVA with Bonferroni's post-test, $^{*}p \leq 0.05$, $^{**}p \leq 0.01$. Errors bars represent SEM; n = 3. (D) Activation of caspase 3/7 in A549 cells following treatment with viruses (100 ppc) or doxorubicin (5 mM). Cells were harvested at 24, 48, and 72 hr, and assayed using the Vybrant FAM Caspase-3 and -7 assay kit.

cell membrane stays intact during the early stages. Early oncosis/necrosis is characterized by propidium iodide (PI) nuclear staining (±Annexin V staining), reflecting compromised plasma membrane integrity. Because the two stains associate with different cellular compartments, it is easy to distinguish cells that are single stained from those that are double stained. Treatment with cisplatin produced features of primary apoptosis (Figure 4A), staining strongly for Annexin V (green) with virtually no membrane leakage (PI, red). Similarly treatment with Ad5 showed a relatively low PI signal and many cells were positive only for Annexin V, presumably undergoing an apoptosis-like death process with intact cell membranes. Few Ad5-killed cells were positive only for PI, suggesting little primary necrosis/oncosis. In contrast, cells treated with EnAd or Ad11p showed extensive PI permeability with some Annexin V staining, a pattern associated mainly with oncosis.

The influence of time on Annexin V and PI staining was studied using flow cytometry (48, 72, and 84 hr post-infection [Figure 4B]). By 72 hr post-infection, cells treated with EnAd showed 35% membrane permeabilization (PI-positive), although by 84 hr post-infection many cells had died and the remainder were 68% positive for PI (±Annexin V). Ad11p gave a similar profile, although the percentage of PI-only cells was less than with EnAd. Ad5 cytotoxicity was only obvious from 84 hr post-infection onward, mainly associated

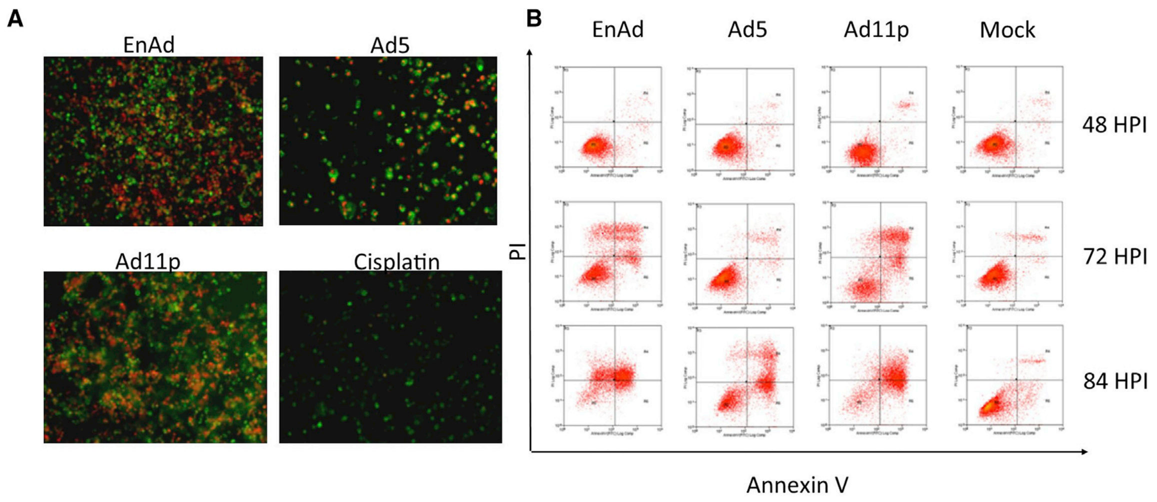

Figure 4. Cells Infected with EnAd Show Plasma Membrane Permeabilization

A549 cells were infected with EnAd (10 ppc), Ad11p (25 ppc) and Ad5 (100 ppc), unless otherwise specified, and data are expressed as a percentage of control mock-treated cells. (A) Annexin V/PI dual staining of the treated cells, observed by fluorescence microscopy (10× magnification). Annexin V was labeled with FITC (green); PI signal indicates lack of plasma membrane integrity (red). Virus-treated cells were imaged at day 3 POI, cisplatin-treated cells were imaged after 24 hr. Mock-treated cells showed only low signals (see Supplemental Information). (B) Flow cytometry analysis of Annexin V/PI time course for virus oncolysis.

with Annexin V positivity with few cells staining only for PI. As controls cisplatin (59% Annexin V staining only) and H_2O_2 (90% PI-positive ± Annexin V) showed distinct staining patterns thought to represent apoptosis and oncosis/necrosis, respectively.

ATP Levels

An important biochemical event leading to oncosis, as opposed to apoptosis, is a rapid fall in intracellular ATP.[17] To maximize relevance to the living situation, cells were cultured in a physiologically relevant level of glucose (5.5 mM) to encourage a realistic metabolic profile. A549 cells infected with Ad5, Ad11p, and EnAd all showed a transient early increase in intracellular ATP, perhaps reflecting adenovirus infection stimulating cell metabolism to facilitate virus production, for example by stimulation of the PI3 kinase pathway by the early virus protein E4ORF1[18] (Figures 5A and 5B). Cells infected with EnAd subsequently show a rapid drop in intracellular ATP levels over a very short period of time (72–96 hr post-infection for cells infected with 5 ppc EnAd [Figure 5A] or 72–84 hr post-infection for 10 ppc EnAd [Figure 5B]). Cells treated with equivalent MOIs of the wild-type Ad11p and Ad5 cause a more gradual decline of ATP (from around 60 to 132 hr post-infection). Lieberthal et al.[19] showed that, if decreases in ATP are less than 75%–85%, cells will either die by apoptosis or survive if mitochondrial function can be restored. However, if ATP depletion is greater than 75%–85%, cells die by a rapid apoptosis-independent ischemic death mechanism. The dashed horizontal line on each graph indicates the 80% threshold for each experiment. The rapid depletion of cellular ATP by EnAd may be a consequence of its strikingly high level of DNA synthesis, where it produced over twice as many virus genomes as Ad11p and over five times more than Ad5 (Figure 5E). Interestingly, simultaneous with the fall in intracellular ATP, EnAd-treated cells also showed a rise in extracellular ATP (Figure 5F), perhaps reflecting increased membrane permeability and non-specific leakage.

Intracellular Calcium

We measured the levels of intracellular calcium in MCF7 and A549 cells infected with EnAd, Ad5, or Ad11p at equal virus particles per cell. Late in the virus infection cycle, intracellular Ca^{2+} levels were observed to increase with all three viruses; however, there is a much greater rise in the case of EnAd than with Ad11p or Ad5 (Figures 5C, 5D, and S7).

The sudden fall in intracellular ATP (reaching levels reported to trigger ischemic cell death), rise in intracellular calcium, and loss of membrane function, coupled with the single BCD noted earlier, all indicate that EnAd infection results in the type of cell death known as oncosis. Death by oncosis is associated with the release of several immunogenic signals from dying cells, providing a pro-inflammatory environment.

EnAd-Mediated Cell Death Causes the Release of Pro-inflammatory and Pro-phagocytic Markers

Apoptotic cells disintegrate into apoptotic bodies with intact membranes, generally regarded as a non-inflammatory mode of death. In contrast, oncosis leads to release of cellular contents and has been implicated as an inflammatory cell death pathway leading to an increase in pro-inflammatory signaling.

HSP70 (heat shock protein 70) and HMGB1 (high-mobility group Box-1) represent endogenous danger signals, and their release from cells is typical of inflammatory cell death.[20,21] Release of HSP70 can be triggered by agents inducing inflammatory cell death such as

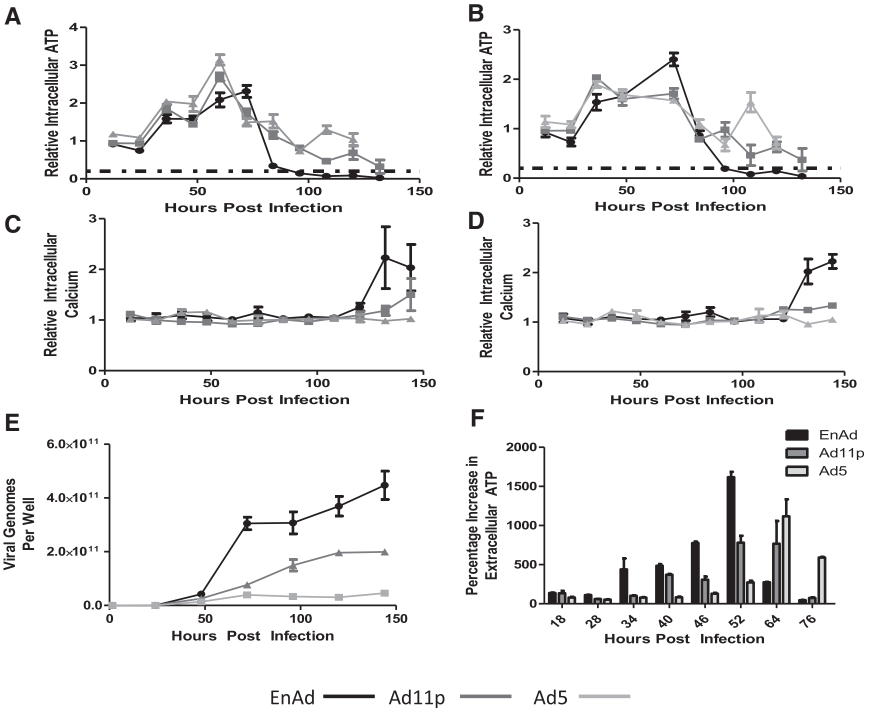

Figure 5. Effects of EnAd Infection and Replication on ATP and Calcium Levels

Effects on cellular ATP levels: A549 cells were grown for 24 hr under low glucose (5 mM) conditions, and treated with viruses at 10 ppc (A) or 5 ppc (B). ATP levels were measured at different times after infection and expressed as percentage of the initial value. Intracellular calcium levels (C and D) were measured in A549 cells grown in normal glucose and expressed as a percentage of the initial value. (E) A549 cells were treated with viruses (100 ppc) for 90 min, washed, and reincubated. Virus genomes were quantified at different times using qPCR. (F) ATP release from A549 cells infected with EnAd (10 ppc), Ad11p (25 ppc), and Ad5 (100 ppc) at different times after infection. Error bars represent SEM; n = 3.

high temperature (60°C) or H_2O_2[22] (used as positive controls [Figure 6C]). Dot blotting showed the release of HMGB1 from cells treated with EnAd and Ad11p started at 3 days post infection (POI), reaching a maximum at day 3.5–4 (Figure 6D), whereas Ad5-treated cells gave only a weak signal even at day 4, when death of most cells was verified by MTS and xCELLigence (Figures 1B–1E). All the cytotoxic agents studied caused some time-dependent release of HSP70 from cells, although EnAd treatment caused significantly greater release than Ad5 and cisplatin, suggesting a more inflammatory death pathway.

Calreticulin exposure on cell surfaces is recognized by low-density lipoprotein-receptor-related protein (LRP) and C1q on phagocytes, essentially acting as an "eat me" signal when expressed on cells. Treatment of A549 (or MCF7) cells with EnAd lead to substantial externalization of calreticulin on the cell surface as measured by flow cytometry (over 80% of cells were positive), higher than that observed when using the wild-type viruses Ad11p and Ad5 (Figure 6E).

To assess potential pro-inflammatory activities in clinical-relevant tissues, we incubated viruses with living "slice" cultures of freshly resected human colorectal cancer liver metastases maintained ex vivo. This model system provides a good surrogate for clinical activity of viruses within the complex multicellular and three-dimensional architecture of human disease. Measured after 72 hr, treatment with EnAd mediated release into the supernatant of TNF-α and IL-6 at significantly higher levels than Ad5, Ad11p, or cisplatin (Figures 6F and 6G). Taken together, these data suggest that the possible pro-inflammatory killing mechanism of EnAd is not limited to cells in culture and may play an important role in the clinical setting too.

EnAd Activates Dendritic Cells and Improves T Cell Stimulation in a Mixed Lymphocyte Reaction

Monocytes isolated from PBMC were briefly cultured (72 hr) in medium containing recombinant human IL-4 and granulocyte-macrophage colony-stimulating factor (GM-CSF) proteins to produce an immature dendritic cell (DC) phenotype. A549 cells were infected with either EnAd or Ad5 (100 ppc), and infection medium was replaced (after 18 hr) with medium containing monocyte-derived DCs. After 48 hr, co-culture DCs were assayed for maturation by flow cytometry. Co-culture of DCs with EnAd-infected tumor cells, or LPS, but not Ad5-infected tumor cells gave up to 8-fold increase in the level of HLA-DR (a component of MHC class II) (Figure 7A). Similarly, the co-stimulatory antigens CD80 and CD86 showed upregulation by incubation of DCs with tumor cells treated with EnAd but not cells treated with Ad5 (Figures 7B and 7C).

To assess the functional consequences of this DC stimulation, we performed a mixed lymphocyte reaction by adding allogeneic CD4 T cells to the mixture of DC, virus, and tumor cells after 48 hr. Under these circumstances, a subset of the T cells are activated by interacting via their T cell receptors with mismatched MHC class I and II molecules, in an antigen-independent manner. In the absence of DCs (i.e., tumor cells, virus, and T cells only) only low levels of T cell activation were seen (Figure 7D). When EnAd-infected tumor cells were incubated with DCs and T cells, the T cells showed a high level of activation as measured by production of IL-2, gamma-interferon, and CD69. Intriguingly, the levels of activation were much higher for tumor cells infected with EnAd than for tumor cells infected with Ad5. These data support the potential for EnAd-mediated tumor cell lysis to create a potent pro-inflammatory environment that can stimulate an adaptive immune response.

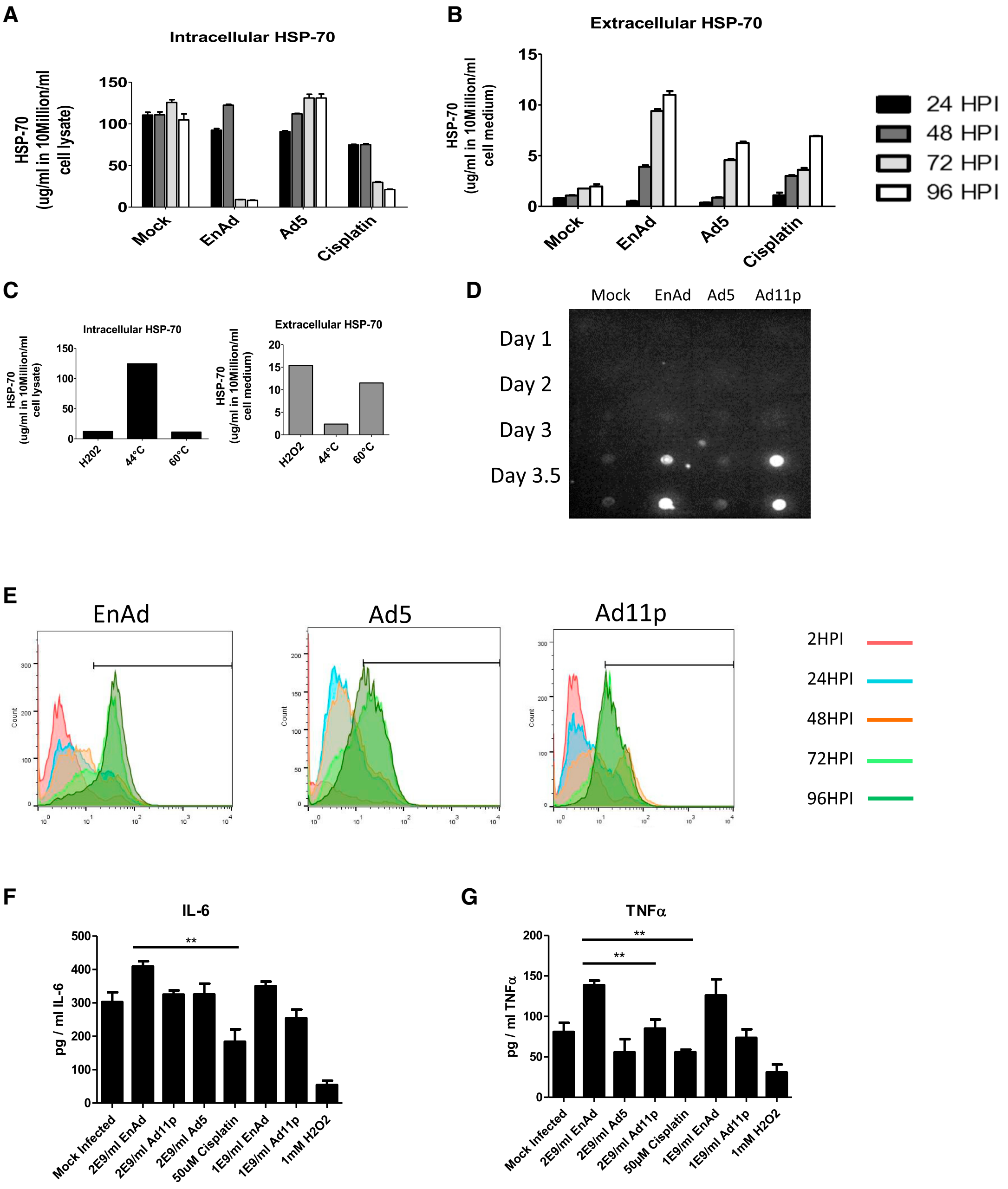

Figure 6. Assessment of the Ability of EnAd to Mediate Release of Pro-inflammatory Cytokines from Clinical Tumor Biopsies

(A–C) Quantification by ELISA of intracellular (A) and extracellular (B) HSP70 protein in supernatants of cells infected with different viruses, as shown. (C) shows temperature-induced positive controls. Significance was assessed by one-way ANOVA, and Tukey's multiple-comparison test, $^*p \leq 0.05$, $^{**}p \leq 0.01$. Error bars represent SEM; n = 3. (D) Dot blot for presence of HMGB1 protein in the supernatants of cells infected with different viruses. (E) Expression of calreticulin in A549 cells following treatment with viruses (100 ppc). Cells were harvested at 24, 48, 72, 96, and 120 hr post-infection (HPI), fixed in formalin, and exposed to an anti-calreticulin antibody, and then analyzed using flow cytometry. The y axis represents the mean expression of calreticulin (FL1). (F and G) Freshly resected human colorectal cancer biopsies were cultured ex vivo and treated immediately with virus particles (2×10^9 virus particles/well) or drugs (50 μM cisplatin or 1 mM H_2O_2). Levels of TNF-α and IL-6 were measured in the supernatant after 72 hr, with significance determined using one-way ANOVA, with Tukey's multiple-comparison test. $^{**}p \leq 0.01$. Error bars represent SEM; n = 3.

DISCUSSION

Lytic viruses are emerging as an important new approach to cancer therapy, providing the possibility of a therapeutic agent that amplifies itself within the target tissue while exerting cytotoxicity selectively to infected cancer cells. In addition to their self-amplifying properties, some oncolytic viruses offer a potentially important advantage over

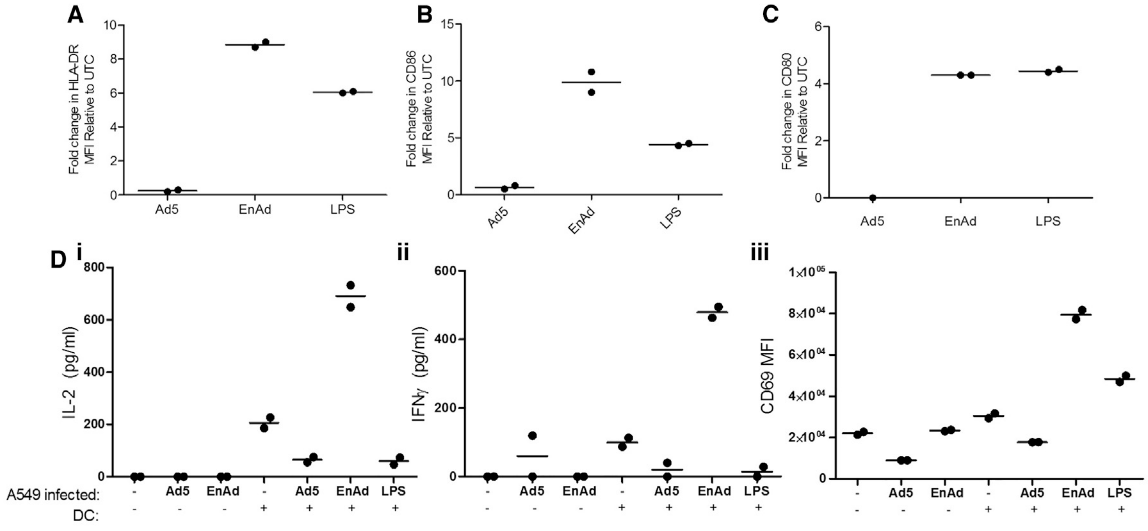

Figure 7. Effect of EnAd-Infected Tumor Cells on Monocyte-Derived Dendritic Cells and CD4 T Cells in a Mixed Tumor-Leukocyte Reaction

Monocytes were positively isolated for CD14 (CD14 MicroBeads; Miltenyi) by MACS from human peripheral blood mononuclear cells. Freshly isolated monocytes were cultured over 3 days in differentiation media, containing recombinant human IL-4 and GM-CSF proteins. A549 cells were grown to 80% confluence and infected with either EnAd or Ad5 at 100 ppc or left untreated. After 18 hr, infection media were removed and cells were washed twice in PBS before adding early monocyte-derived dendritic cells (DCs). (A–C) After 48 hr co-culture, DCs were assayed for maturation by flow cytometry for HLA-DR (A), CD86 (B), and CD80 expression (C) or co-cultured with enriched allogeneic human CD4 T cells (CD4 MicroBeads; Miltenyi), obtained from fresh PBMCs by MACS. (D) T cell activation was assessed 24 hr later by ELISA (eBioscience) of co-culture supernatants for IL-2 (i) and IFN-γ (ii) production or CD69 (iii) by flow cytometry (NxT Attune; LifeTech). For (A)–(C), each experiment was performed twice, using DCs from different donors, and assessed by flow cytometry. Individual values and means are shown. For (D), each experiment was performed four times, using different mismatched DC and T cells, and data from single representative experiments are shown.

conventional cancer therapeutics by expressing virus proteins that take control of the infected cell and kill it at the appropriate stage of the virus life cycle, to allow release and spread of progeny virions. Such cytocidal mechanisms may exploit pathways of programmed cell death, or may operate largely independent of conventional cellular death pathways. For example, cell lysis might be expected to maximize opportunities for virion spread, although it could also expose cellular components that would stimulate a pro-inflammatory immune environment. Some viruses may also be engineered to encode immunomodulatory biologics, and express them selectively within the tumor microenvironment. This range of attractive anti-cancer mechanisms can in principle be combined within single-virus therapeutics and form the mechanistic basis of the rapidly burgeoning concept known as "oncolytic vaccines."

Not all virus-mediated killing is pro-inflammatory. Many "lytic" viruses have evolved to usurp intrinsic cellular death pathways to mediate killing at the appropriate time in the virus cycle.[23] Several of these intrinsic pathways (such as apoptosis) are designed to be non-inflammatory or even immune suppressive,[24,25] perhaps allowing the virus to maximize spread and propagation before immune eradication occurs. In the studies reported here, the extended time course of killing by Ad5, coupled with its limited release of inflammatory mediators, might suggest that it exploits some aspects of cellular death pathways, although the lack of caspase 3 activation indicates neither intrinsic nor extrinsic apoptosis pathways are fully engaged. Other groups have previously reported that Ad5 kills by autophagy and autophagy-triggered caspase activity,[26] and that overexpression of the adenovirus death protein (ADP) accelerates apoptosis.[27] Still others report that Ad5 viruses lacking E3 (and therefore lacking ADP) are less virulent than those with ADP and kill cells by a process related to necrosis,[28,29] and there is at least one report that death is via a programmed pathway distinct from both apoptosis and necrosis, independent of ADP status of the virus.[30] It seems likely that the precise mechanism of virus cytotoxicity may vary between cell types, but it also seems reasonable to surmise that the absence of ADP (or any obvious homolog) from Ad11p and EnAd may contribute to their virulence and necrosis-like death mechanism.

EnAd appears to kill cells by a process similar to that known as "oncosis," or ischemic cell death. Majno and Joris[31] describe oncosis as a form of accidental cell death accompanied by cellular swelling, organelle swelling, blebbing, and increased membrane permeability caused by the failure of the ionic pumps of the plasma membrane. Trump et al.[17,32] associate oncosis (blister formation) with increases in concentrations of cytosolic calcium and rearrangement of cytoskeletal proteins.[17] The most characteristic distinguishing feature of oncosis is the formation of large single membrane blisters at the time of death, presumably a result of loss of control of cellular ion pumps leading to sudden osmotic swelling. This feature was most obvious and dramatic in the time-lapse videos (Movies S1, S2, S3, S4, and S5) but can also be seen in Figure 1L. Cells infected with

EnAd also showed many other features attributed to oncosis, including leaky plasma membranes, release of ATP, HMGB1, and HSP70, externalization of calreticulin and calcium influx, together with few features of either apoptosis or necroptosis.

The specificity of EnAd for human cells severely restricts the animal models available for preclinical testing of immunogenicity. Xenograft models in nude and SCID mice are limited to studies of innate immunity and the use of fully humanized NRG mice is expensive and clinical relevance is largely untested. We therefore used ex vivo culture of thinly sliced fresh human tumor biopsies as a superior model to monitor the performance of human-specific agents within the complex microenvironment of human tumors. Under these conditions, EnAd showed increased induction of proinflammatory cytokines TNF and IL-6 that further support its development in clinical trials. To supplement these observations, we performed in vitro studies using blood cells from human donors to assess the functional consequences of EnAd-mediated tumor cell lysis on antigen presentation. Under these conditions, EnAd-infected tumor cells showed activation of dendritic cells far more than Ad5. In a mixed tumor-leukocyte reaction (to endow basal levels of CD4 T cell activation), the EnAd-infected tumor cells also led to much greater stimulation of CD4 T cells than the other treatments (Figure 7), and this suggests that EnAd may be particularly suited to creating an adaptive immune response in human clinical trials.

Apart from its pro-inflammatory properties, oncosis occurs largely independent of cellular programmed death pathways and may therefore provide a means to overcome drug resistance pathways that are based on deficient apoptosis mechanisms (S.I., Y.D., M. Bauzon, J. Lei., M. Duffy., S. Alvis, B.C., A. Lieber, T.H., L.W.S., J.B., and K.D.F., unpublished data). EnAd was bioselected from a library of chimeric adenoviruses, on the basis of its ability to infect cancer cells, replicate, and then escape quickly from them. As such, it has not been subject to evolution in the wild and has not evolved to fill a sustainable niche. Its life cycle is shorter (and associated cytotoxicity faster) than either of its parental viruses (Ad11p and Ad3) and much shorter than Ad5. In part, this probably reflects its smaller genome size (32,325 bp compared to 34,794 bp, through elimination of several E3 genes), although it might also reflect the chimeric E2B region and small deletion in E4ORF4.[5] Encoded in E2B is a chimeric DNA polymerase, with DNA binding regions thought to recognize the Ad11p ITRs efficiently,[5] and this may well contribute to the large number of EnAd genomes produced in infected cells (more than twice as many as Ad11p and five times more than Ad5). E4ORF4, which is missing in EnAd, normally (in Ad5) has several roles including feedback control of E1A and regulating spliceosome activity that may "balance" early and late virus expression as well as deregulating activity of AMPK, the cellular energy sensor. It is tempting to consider that EnAd, without E4ORF4, may show a less balanced life cycle, and its high level of genome synthesis may rapidly consume cellular resources and contribute to a catastrophic fall in ATP levels that could trigger the onset of the oncosis-like cell death observed.

Group B adenoviruses based on Ad11p offer enticing potential as virotherapy agents. Necrotic death is a goal of many cancer biologists as it can avoid apoptosis pathways[33] and simultaneously causes release of pro-inflammatory mediators.[34] These viruses also offer several advantages in the context of improved delivery to tumors. For example, the receptors CD46 and desmoglein 2 are upregulated on many carcinoma cells,[35,36] but also most humans have only low levels of pre-existing neutralizing antibodies to Ad11.[37] In contrast to Ad5-based virotherapy agents, which are easily neutralized by human blood, Ad11p-based viruses such as EnAd are far less susceptible to neutralization and even offer systemic bioavailability.[38] In the case of EnAd, these properties are combined with a relatively short life cycle that should allow rapid spread from cell to cell, maximizing tumor cell killing before a neutralizing antiviral response is produced. Finally, the possibility of "arming" such viruses to express immune-stimulatory transgenes in situ should embody the full potential of an oncolytic vaccine approach, where virus activity combines with the secreted transgene product to create sufficient immune provocation to engender an anticancer immune response.[39]

MATERIALS AND METHODS

Chemicals

Pifithrin-α and ZVAD-fmk were from Tocris Bioscience. All other chemicals were from Sigma-Aldrich.

Cell Lines

Human A549 lung adenocarcinoma and SKOV-3 ovarian carcinoma, DLD-1 adenocarcinoma, IGROV ovarian carcinoma, and MCF7 breast adenocarcinoma cell lines (ATCC) were grown in DMEM containing 2 mM glutamine, 10% FCS, and 50 μg/mL penicillin-streptomycin (P/S) (PAA). H1299 cells (gift from Jaroslav Zak, Ludwig Institute, Oxford, United Kingdom) were co-transfected with pTET-on plasmid and tet-responsive plasmid containing wild-type p53 and pBabe puro (selectable marker). Tissue culture plastic ware was from Corning. Cells for all studies were seeded at 10,000 cells/well (96-well plate) 24 hr before use, unless otherwise indicated.

Adenoviruses

EnAd was from PsiOxus Therapeutics, and wild-type Ad3, Ad5, Ad11p, and ONYX-015 were from The Native Antigen Company, all propagated on HEK293 cells and purified twice on CsCl gradients. Virus concentration (virus particles [VPs] per milliliter) was determined using Picogreen assay (Invitrogen) to measure DNA. Purified bacteriophage λ DNA was used as standard, and particle number was calculated by adenovirus genome molecular weight.

Infectious particles were measured by $TCID_{50}$ assay on A549 cells using 1:10 serial virus dilutions. Plates were observed day 7 POI and stained with formaldehyde-crystal violet. The highest dilutions showing cytopathic effect (CPE) were counted, and infectious particle unit (plaque-forming units [PFUs] per milliliter) calculated using the Karber formula.

Cell Viability and Cytotoxicity Assays

Cells were seeded as above and incubated with serial virus dilutions. After 5 days, MTS reagent (20 μL) was added and color change was read at 490 nm (CellTiter 96 aqueous non-radioactive cell proliferation assay; Promega). Plates were fixed in 4% paraformaldehyde, stained with 0.1% crystal violet, and then scanned with a CannonScan 44000F scanner.

Real-time monitoring of cell growth used the xCELLigence RTCA DP instrument (Roche). Exponentially growing cells were treated 24 hr after seeding (time zero on the graphs), and the impedance of each well was subsequently monitored automatically every 15 min and expressed as a CI.

Electron Microscopy

The cells were gently trypsinized and collected by centrifugation, followed by fixing in 2.5% glutaraldehyde and post-fixing in 2% osmium tetroxide (both in 0.1 M cacodylate buffer), and were treated en bloc with 2% uranyl acetate in distilled water. Samples were dehydrated and treated with propylene oxide prior to embedding in Spurr's epoxy resin. The 1-μm sections stained with Azure A were examined by light microscopy to identify areas of interest. Thin sections were cut and stained with uranyl acetate and lead citrate for examination in a Jeol 1200EX electron microscope.

SDS-PAGE, Western Blot, and Dot Blot

Cell pellets (600 × *g*, 5 min) were lysed with RIPA buffer containing protease inhibitors (Roche). Supernatants were adjusted to 2 mg protein/mL and denatured (95°C, 5 min), and 20 μL was loaded onto the gel. After SDS-PAGE (10%, 120 V) and transfer to nitrocellulose (GE Healthcare; 300 mA, 4 hr), the membrane was blocked with 5% milk in PBST (PBS with 0.1% Tween 20), and then incubated with primary antibody overnight at 4°C. The membrane was incubated with secondary antibody for 1 hr, stained with ECL (GE Healthcare), and recorded using a digital system with Fluorochem 8900 software.

Dot blots used 1-μL samples on nitrocellulose membranes and were processed as above.

Primary antibodies were mouse monoclonals: IgG anti-β-actin (AC-74) (A2228; Sigma), IgG1 anti human p53 (2C3) (MCA35102; AbD Serotec), IgG anti-HMGB1 (H9664; Sigma), and polyclonal anti-human HSP 70 antibody (841680; R&D Systems). Dilutions were 1:1000. Secondary antibodies were as follows: goat anti-mouse IgG HRP (Promega) (1:5,000 dilution). SDS-PAGE gels were directly visualized with Instant Blue kit (Expedeon).

Cell Cycle Analysis

The treated cells were trypsinized and pelleted, fixed, permeabilized, and stained with 100 μg/mL PI and 100 μg/mL RNase A (Sigma) overnight, prior to flow cytometry (FACSCalibur, BD Biosciences with Cell QuestPro software). Cells in the four phases (percentage) were calculated on the basis of DNA content.

Measurement of Caspase 3/7 Activation

Cells were harvested after various lengths of treatment and stained with Vybrant FAM Caspase-3 and -7 assay kit (Thermo Fisher). Stained cells were suspended in 4% formaldehyde, resuspended in MACS buffer, and evaluated by flow cytometry as above using FlowJo software (Tree Star).

Measurement of ATP and Calcium Levels

ATP in virus-treated cells was measured using the Intracellular ATP kit HS (Biothema) by the manufacturer's instructions. Samples were analyzed by luminometry, normalized for protein, and expressed as percentage of controls. Intracellular calcium levels were measured using the fluo-8 calcium assay (AbCam). The dye was added to cells and incubated for 1 hr, and then fluorescence was measured at excitation/emission of 490/525 nm.

Production of Monocyte-Derived Dendritic Cells

Monocytes were positively isolated by magnetic-activated cell sorting (MACS) for CD14 (CD14 MicroBeads; Miltenyi) from human peripheral blood mononuclear cells (PBMCs), obtained by Ficoll gradient. Freshly isolated monocytes were cultured for 3 days in differentiation media, containing recombinant human IL-4 and GM-CSF proteins (R&D Systems) to produce DCs.

Mixed Tumor-Leukocyte Reaction Assay

A549 cells were grown to 80% confluence and treated with either EnAd or Ad5 at 100 ppc or left untreated. After 18 hr, infection media were removed and cells washed twice in PBS before adding monocyte-derived DCs. After 48 hr, co-culture DCs were assayed for maturation by flow cytometry or co-cultured with enriched allogeneic human CD4 T cells (CD4 MicroBeads; Miltenyi), obtained from fresh PBMCs by MACS. T cell activation was assessed 24 hr later by flow cytometry (NxT Attune; LifeTech) or IL-2 and IFN-g (eBioscience) production by ELISA of culture supernatants.

Annexin V/PI Stain

Cells were treated as for cell-cycle analysis, stained with Annexin V-fluorescein and PI (each 1%) from the Annexin V/PI kit (Roche), and evaluated using Zeiss Axiovert 25 Inverting light/fluorescence microscope, and images were recorded by Nikon DS-U2 camera and processed with NIS-element AR 3.00 software.

For flow cytometry, cells were labeled as above, diluted to 500 μL, measured using a Dako Cyan flow cytometer (λ_{ex}, 488 nm; λ_{em}, 530/30 nm [FL1, green], 585/42 nm [FL2, red]), and analyzed using Summit 4.3 software.

Determination of Adenovirus Genomes by qPCR

Samples were extracted with the GenElute mammalian genomic DNA Miniprep Kit (Sigma). Primers and FAM-TAMRA probes (Sigma) recognized the fiber gene. Analysis of Ad11p and EnAd used forward primer TACATGCACATCGCCGGA, reverse CGGGCGAACTG CACCA, and probe CCGGACTCAGGTACTCCGAAGCATCCT;

Ad5 analysis used forward primer TGGCTGTTAAAGGCAGT TTGG, reverse GCACTCCATTTTCGTCAAATCTT, and probe TCCAATATCTGGAACAGTTCAAGTGCTCATCT. Primers and probe were added to QPCRBIo probe mix Hi-Rox (PCR Biosystems) master mix. The qPCR was run on an ABI PRISM 7000 (Applied Biosystems); details of setup and data analysis have been reported before.[26]

ELISA

HSP70 was quantified by DuoSet IC Human/Mouse/Rat total HSP70 ELISA kit (R&D Systems), and IL-6 and TNF-α were quantified by mini ELISA kits (Peprotech), all according to manufacturers' instructions.

Ex Vivo Biopsy Culture

Tumor samples were collected immediately post-surgery from consented patients on ice (Central Office of Research Ethics Committees, permission C02.285). HarrisCore tissue corers and scalpels were used to produce multiple uniform tissue slices (thickness: 1 mm; diameter: 5 mm). Sections were then incubated in 24-well plates in RPMI full growth medium (2.5 mL) at 37°C. Experimental treatments started immediately, with addition of viruses or drugs. After 3 days, supernatants were stored for cytokine ELISA while tissues were fixed with 4% paraformaldehyde and transferred into 80% ethanol for paraffin embedding and sectioning.

Statistical Analysis

Mean values are shown ± SD or SEM as indicated. Data were compared between different groups using two-sample (unpaired) t test assuming equal variance or one-way ANOVA followed by Tukey's multiple-comparisons test.

SUPPLEMENTAL INFORMATION

Supplemental Information includes seven figures, one table, and five movies and can be found with this article online at http://dx.doi.org/10.1016/j.omto.2016.11.003.

AUTHOR CONTRIBUTIONS

K.D.F., A.B., J.B., T.H., A.D., B.C., and L.W.S. conceived and designed the study. A.D., Y.D., H.C., G.K., D.B., A.T., S.I., P.J., S.L.C., and D.J.P.F. conducted the experiments. M.A.S. provided primary tumor material from his surgery. L.W.S., A.D., Y.D., and K.D.F. wrote the manuscript with input from all the authors.

CONFLICTS OF INTEREST

Some of the authors (A.B., B.C., J.B., T.H., K.D.F., and L.W.S.) own equity or share options in PsiOxus and some are employed by the company.

ACKNOWLEDGMENTS

We are grateful to Cancer Research UK for support (Programme Grant C552/A17720) and to Pav Gill, Joy Wiles, Gemma Marsden, and Bhumika Patel for facilitating tissue sample collection.

REFERENCES

1. Russell, S.J., Peng, K.W., and Bell, J.C. (2012). Oncolytic virotherapy. Nat. Biotechnol. *30*, 658–670.
2. Seymour, L.W., and Fisher, K.D. (2016). Oncolytic viruses: finally delivering. Br. J. Cancer *114*, 357–361.
3. Heo, J., Reid, T., Ruo, L., Breitbach, C.J., Rose, S., Bloomston, M., Cho, M., Lim, H.Y., Chung, H.C., Kim, C.W., et al. (2013). Randomized dose-finding clinical trial of oncolytic immunotherapeutic vaccinia JX-594 in liver cancer. Nat. Med. *19*, 329–336.
4. Andtbacka, R.H., Kaufman, H.L., Collichio, F., Amatruda, T., Senzer, N., Chesney, J., Delman, K.A., Spitler, L.E., Puzanov, I., Agarwala, S.S., et al. (2015). Talimogene laherparepvec improves durable response rate in patients with advanced melanoma. J. Clin. Oncol. *33*, 2780–2788.
5. Kuhn, I., Harden, P., Bauzon, M., Chartier, C., Nye, J., Thorne, S., Reid, T., Ni, S., Lieber, A., Fisher, K., et al. (2008). Directed evolution generates a novel oncolytic virus for the treatment of colon cancer. PLoS One *3*, e2409.
6. Wang, H., Li, Z.Y., Liu, Y., Persson, J., Beyer, I., Möller, T., Koyuncu, D., Drescher, M.R., Strauss, R., Zhang, X.B., et al. (2011). Desmoglein 2 is a receptor for adenovirus serotypes 3, 7, 11 and 14. Nat. Med. *17*, 96–104.
7. O'Shea, C.C., Choi, S., McCormick, F., and Stokoe, D. (2005). Adenovirus overrides cellular checkpoints for protein translation. Cell Cycle *4*, 883–888.
8. Scrace, S., O'Neill, E., Hammond, E.M., and Pires, I.M. (2013). Use of the xCELLigence system for real-time analysis of changes in cellular motility and adhesion in physiological conditions. Methods Mol. Biol. *1046*, 295–306.
9. Weerasinghe, P., Hallock, S., Tang, S.C., and Liepins, A. (2001). Role of Bcl-2 family proteins and caspase-3 in sanguinarine-induced bimodal cell death. Cell Biol. Toxicol. *17*, 371–381.
10. Weerasinghe, P., and Buja, L.M. (2012). Oncosis: an important non-apoptotic mode of cell death. Exp. Mol. Pathol. *93*, 302–308.
11. Hallock, S., Tang, S.C., Buja, L.M., Trump, B.F., Liepins, A., and Weerasinghe, P. (2007). Aurintricarboxylic acid inhibits protein synthesis independent, sanguinarine-induced apoptosis and oncosis. Toxicol. Pathol. *35*, 300–309.
12. Querido, E., Blanchette, P., Yan, Q., Kamura, T., Morrison, M., Boivin, D., Kaelin, W.G., Conaway, R.C., Conaway, J.W., and Branton, P.E. (2001). Degradation of p53 by adenovirus E4orf6 and E1B55K proteins occurs via a novel mechanism involving a Cullin-containing complex. Genes Dev. *15*, 3104–3117.
13. Forrester, N.A., Sedgwick, G.G., Thomas, A., Blackford, A.N., Speiseder, T., Dobner, T., Byrd, P.J., Stewart, G.S., Turnell, A.S., and Grand, R.J. (2011). Serotype-specific inactivation of the cellular DNA damage response during adenovirus infection. J. Virol. *85*, 2201–2211.
14. Sohn, D., Graupner, V., Neise, D., Essmann, F., Schulze-Osthoff, K., and Jänicke, R.U. (2009). Pifithrin-alpha protects against DNA damage-induced apoptosis downstream of mitochondria independent of p53. Cell Death Differ. *16*, 869–878.
15. McIlwain, D.R., Berger, T., and Mak, T.W. (2015). Caspase functions in cell death and disease. Cold Spring Harb. Perspect. Biol. *7*, a026716.
16. Buja, L.M. (2005). Myocardial ischemia and reperfusion injury. Cardiovasc. Pathol. *14*, 170–175.
17. Trump, B.F., Berezesky, I.K., Chang, S.H., and Phelps, P.C. (1997). The pathways of cell death: oncosis, apoptosis, and necrosis. Toxicol. Pathol. *25*, 82–88.
18. Kong, K., Kumar, M., Taruishi, M., and Javier, R.T. (2014). The human adenovirus E4-ORF1 protein subverts discs large 1 to mediate membrane recruitment and dysregulation of phosphatidylinositol 3-kinase. PLoS Pathog. *10*, e1004102.
19. Lieberthal, W., Menza, S.A., and Levine, J.S. (1998). Graded ATP depletion can cause necrosis or apoptosis of cultured mouse proximal tubular cells. Am. J. Physiol. *274*, F315–F327.
20. Scaffidi, P., Misteli, T., and Bianchi, M.E. (2002). Release of chromatin protein HMGB1 by necrotic cells triggers inflammation. Nature *418*, 191–195.
21. Basu, S., Binder, R.J., Suto, R., Anderson, K.M., and Srivastava, P.K. (2000). Necrotic but not apoptotic cell death releases heat shock proteins, which deliver a partial maturation signal to dendritic cells and activate the NF-kappa B pathway. Int. Immunol. *12*, 1539–1546.

22. Brusa, D., Migliore, E., Garetto, S., Simone, M., and Matera, L. (2009). Immunogenicity of 56 degrees C and UVC-treated prostate cancer is associated with release of HSP70 and HMGB1 from necrotic cells. Prostate *69*, 1343–1352.

23. O'Brien, V. (1998). Viruses and apoptosis. J. Gen. Virol. *79*, 1833–1845.

24. Voll, R.E., Herrmann, M., Roth, E.A., Stach, C., Kalden, J.R., and Girkontaite, I. (1997). Immunosuppressive effects of apoptotic cells. Nature *390*, 350–351.

25. Vaux, D.L. (1993). Toward an understanding of the molecular mechanisms of physiological cell death. Proc. Natl. Acad. Sci. USA *90*, 786–789.

26. Jiang, H., White, E.J., Ríos-Vicil, C.I., Xu, J., Gomez-Manzano, C., and Fueyo, J. (2011). Human adenovirus type 5 induces cell lysis through autophagy and autophagy-triggered caspase activity. J. Virol. *85*, 4720–4729.

27. Yun, C.O., Kim, E., Koo, T., Kim, H., Lee, Y.S., and Kim, J.H. (2005). ADP-overexpressing adenovirus elicits enhanced cytopathic effect by induction of apoptosis. Cancer Gene Ther. *12*, 61–71.

28. Abou El Hassan, M.A., van der Meulen-Muileman, I., Abbas, S., and Kruyt, F.A. (2004). Conditionally replicating adenoviruses kill tumor cells via a basic apoptotic machinery-independent mechanism that resembles necrosis-like programmed cell death. J. Virol. *78*, 12243–12251.

29. Glockzin, G., Mantwill, K., Jurchott, K., Bernshausen, A., Ladhoff, A., Royer, H.D., Gansbacher, B., and Holm, P.S. (2006). Characterization of the recombinant adenovirus vector AdYB-1: implications for oncolytic vector development. J. Virol. *80*, 3904–3911.

30. Baird, S.K., Aerts, J.L., Eddaoudi, A., Lockley, M., Lemoine, N.R., and McNeish, I.A. (2008). Oncolytic adenoviral mutants induce a novel mode of programmed cell death in ovarian cancer. Oncogene *27*, 3081–3090.

31. Majno, G., and Joris, I. (1995). Apoptosis, oncosis, and necrosis. An overview of cell death. Am. J. Pathol. *146*, 3–15.

32. Trump, B.F., and Berezesky, I.K. (1992). The role of cytosolic Ca^{2+} in cell injury, necrosis and apoptosis. Curr. Opin. Cell Biol. *4*, 227–232.

33. Hu, X., and Xuan, Y. (2008). Bypassing cancer drug resistance by activating multiple death pathways—a proposal from the study of circumventing cancer drug resistance by induction of necroptosis. Cancer Lett. *259*, 127–137.

34. Iyer, S.S., Pulskens, W.P., Sadler, J.J., Butter, L.M., Teske, G.J., Ulland, T.K., Eisenbarth, S.C., Florquin, S., Flavell, R.A., Leemans, J.C., and Sutterwala, F.S. (2009). Necrotic cells trigger a sterile inflammatory response through the Nlrp3 inflammasome. Proc. Natl. Acad. Sci. USA *106*, 20388–20393.

35. Thorsteinsson, L., O'Dowd, G.M., Harrington, P.M., and Johnson, P.M. (1998). The complement regulatory proteins CD46 and CD59, but not CD55, are highly expressed by glandular epithelium of human breast and colorectal tumour tissues. APMIS *106*, 869–878.

36. Brennan, D., and Mahoney, M.G. (2009). Increased expression of Dsg2 in malignant skin carcinomas: a tissue-microarray based study. Cell Adhes. Migr. *3*, 148–154.

37. Holterman, L., Vogels, R., van der Vlugt, R., Sieuwerts, M., Grimbergen, J., Kaspers, J., Geelen, E., van der Helm, E., Lemckert, A., Gillissen, G., et al. (2004). Novel replication-incompetent vector derived from adenovirus type 11 (Ad11) for vaccination and gene therapy: low seroprevalence and non-cross-reactivity with Ad5. J. Virol. *78*, 13207–13215.

38. Di, Y., Seymour, L., and Fisher, K. (2014). Activity of a group B oncolytic adenovirus (ColoAd1) in whole human blood. Gene Ther. *21*, 440–443.

39. Sivendran, S., Pan, M., Kaufman, H.L., and Saenger, Y. (2010). Herpes simplex virus oncolytic vaccine therapy in melanoma. Expert Opin. Biol. Ther. *10*, 1145–1153.

OMTO, Volume *4*

Supplemental Information

Oncolytic Group B Adenovirus Enadenotucirev Mediates Non-apoptotic Cell Death with Membrane Disruption and Release of Inflammatory Mediators

Arthur Dyer, Ying Di, Hugo Calderon, Sam Illingworth, Gray Kueberuwa, Alison Tedcastle, Phil Jakeman, Suet Lin Chia, Alice Brown, Michael A. Silva, David Barlow, John Beadle, Terry Hermiston, David J.P. Ferguson, Brian Champion, Kerry D. Fisher, and Leonard W. Seymour

Table 1. Particle number and infectious titre of virus stocks

	Particle number/ml (VP/ml)*	Infectious particle number/ml (PFU/ml)*	**VP/PFU ratio**
Ad5	2.50E12	7.07E10	**35.3**
Ad5 (fig 1J, fig 5 and fig 6E)	3.16E11	1.05E10	**30.1**
Ad11	1.90E12	6.13E10	**31.0**
Ad11 (fig 1K, fig 5 and fig 6E)	2.35E12	7.15E10	**32.9**
EnAd	1.65E12	4.77E10	**34.6**
EnAd (fig 1L, fig 5 and fig 6E)	1.69E12	4.81E10	**35.1**
Ad3	1.45E12	ND	**ND**
ONYX-15	4.88E12	ND	**ND**

*VP/ml was measured by Picogreen assay and PFU/ml was measured by 7 days TCID50 assay on A549 cells.

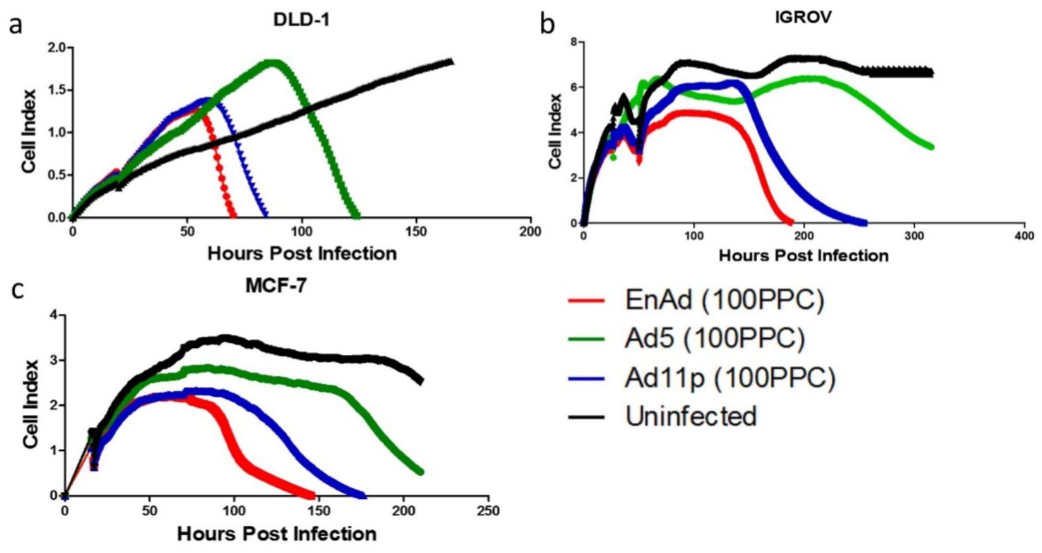

Supplementary figure S1 - Comparison of potency and time course of cytotoxicity of different adenoviruses in different cell lines. DLD-1 **(a)** IGROV **(b)** and MCF-7 **(c)** cell growth and cytotoxicity curve under 100PPC of different adenoviruses, measured by xCelligence cell monitor (Roche)**.**

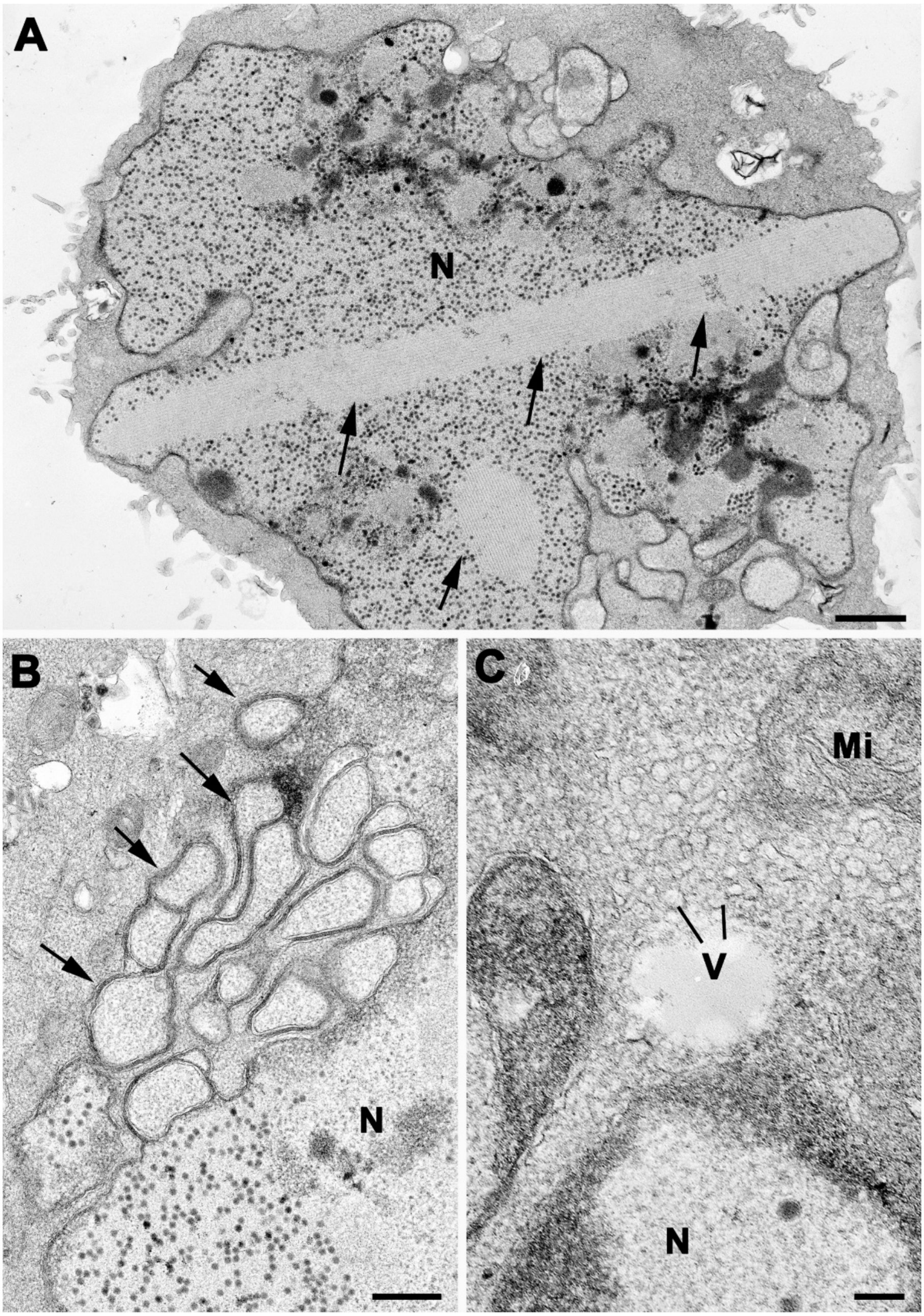

Supplementary Figure S2. Electron micrographs of non-physiological features observed in A549 cells infected with Ad5. Arrows in **A** indicate intranuclear crystals of virus proteins, in **B** excessive folding of the nuclear membrane and in **C** the presence of many vesicles or membranes in the cytoplasm (V). N= nucleus, Mi=mitochondrion. The bars in A - 1um, B - 500nm and C -100nm.

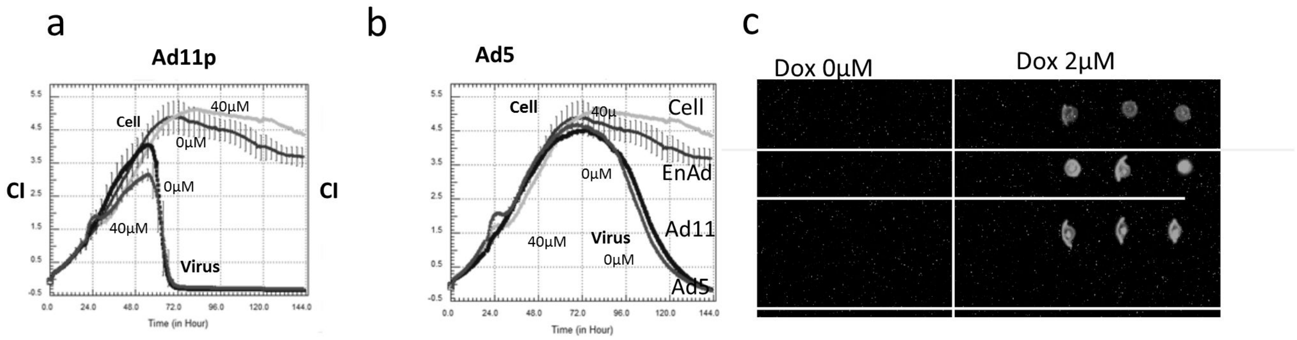

Supplementary Figures S3 a&b The impact of p53 inhibitor Pitfithrin α on activity of Ad5 and Ad11p in A549 cells. Cell growth was assessed using the Xcelligence system as described, adenoviruses were applied at 100 PPC, 24h after cell seeding (i.e. at 24h on the graph). Pitfithrin α was applied at 40 µM, 5 h after viral infection. **c**, Dot blot for p53 of the samples obtained from Fig3 **c** to verify p53 induction by doxycycline. Cells were lysed using 50µl Promega lysis buffer, and 1µl lysate from each well was assayed.

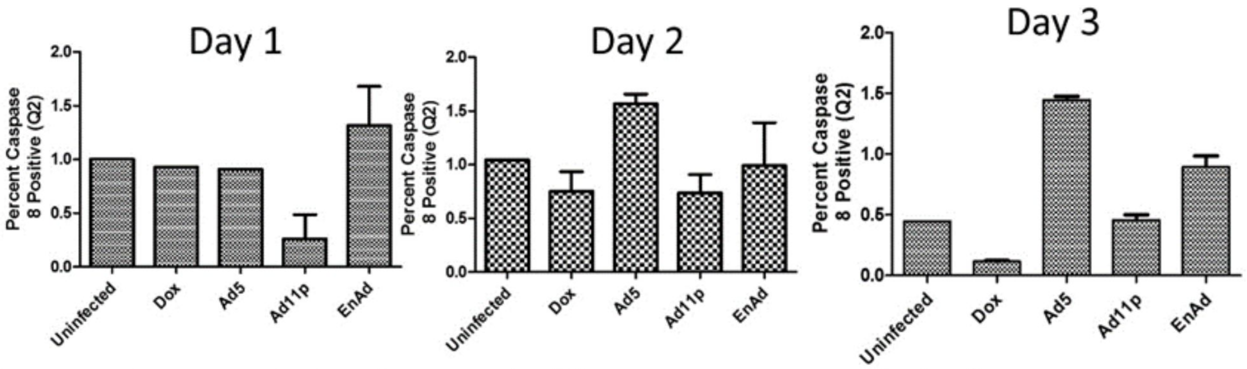

Supplementary Figure S4. Activation of caspase 8 by different treatments, assessed using flow cytometry. Activation of caspase 8 in A549 cells following treatment with viruses (100 ppc) or doxorubicin (5 mM). Cells were harvested at 24, 48 and 72h and assayed using the Vybrant FAM Caspase-8 assay kit.

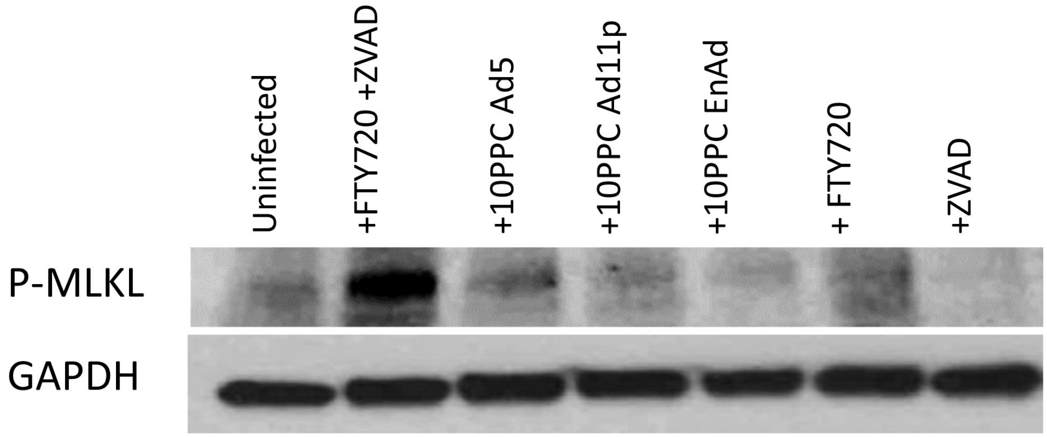

Supplementary Figure S5. Presence of markers of necroptosis during infection with adenoviruses

Western blot analysis of Phospho-MLKL (Ser358) in A549 Cells treated with different viruses and drugs. Cells were treated with 20mMol FTY720, 20µMol ZVAD, a combination of both FTY720 and ZVAD or 10PPC of Ad5, Ad11p or EnAd. 4 days post treatment cells were lysed and analysed via western blot for phospho-MLKL (Ser358) and GAPDH.

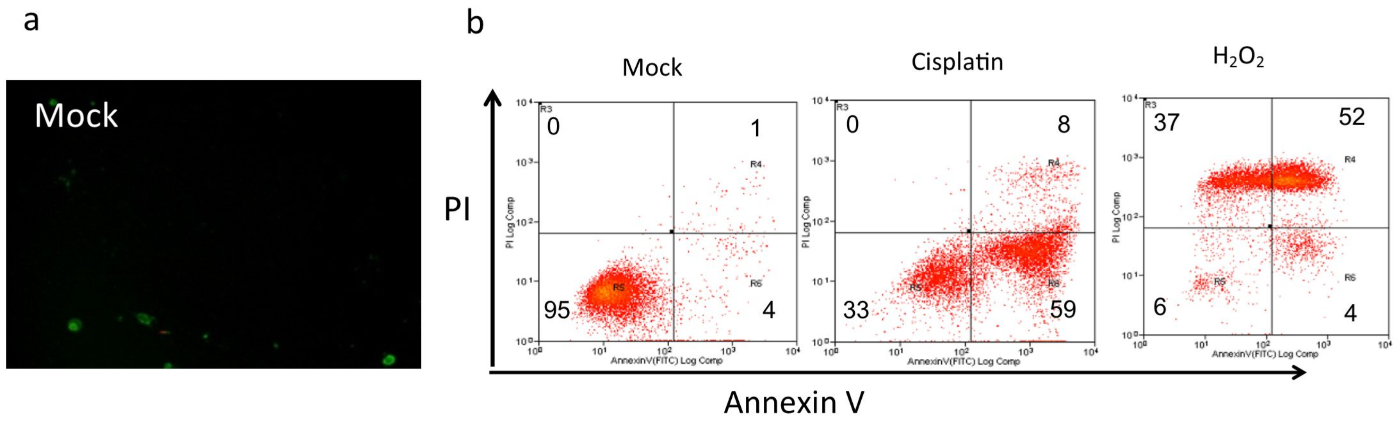

Supplementary Figure S6a. AnnexinV/PI dual staining of non-treated cells. Cells were observed by fluorescence microscopy, with 10X magnification**.** Annexin V was labelled with FITC (green color); PI was shown as red colour. **b**, **Representive Annexin V/PI double staining dot blot analysis by flow cytometry.** Cisplatin was applied at 50µM for 24 h, and H_2O_2 was used at 1mM for 3 h.

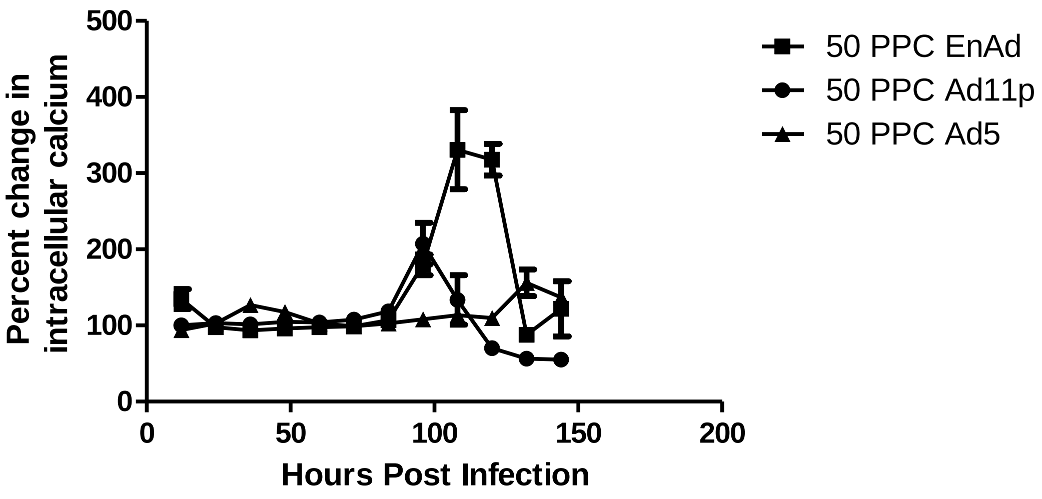

Supplementary figure S7 Effects of EnAd, Ad5 and Ad11p infection and replication on calcium levels in A549 Cells. Intracellular calcium levels were measured in A549 Cells and expressed as % of the initial value
